# Video‐Based Data‐Driven Models for Diagnosing Movement Disorders: Review and Future Directions

**DOI:** 10.1002/mds.30327

**Published:** 2025-08-15

**Authors:** Rafael Martínez‐García‐Peña, Lisette H. Koens, George Azzopardi, Marina A.J. Tijssen

**Affiliations:** ^1^ Department of Neurology University of Groningen, University Medical Centre Groningen (UMCG) Groningen The Netherlands; ^2^ Bernoulli Institute for Mathematics, Computer Science and Artificial Intelligence, University of Groningen Groningen The Netherlands; ^3^ Department of Neurology and Clinical Neurophysiology, Martini Ziekenhuis Groningen The Netherlands; ^4^ Expertise Centre Movement Disorders Groningen, University Medical Centre Groningen (UMCG) Groningen The Netherlands

**Keywords:** artificial intelligence, deep learning, machine learning, movement disorders, video

## Abstract

Movement disorders are abnormal, involuntary movements that can heavily impact a person's quality of life. In clinical practice, diagnosis and severity assessments rely mainly on visual clinical inspections (ie, on subjective expert opinion). With clinical videos commonly acquired as part of examinations, novel data‐driven models have emerged that use machine learning (ML) and deep learning (DL) algorithms to capture human actions and recognize their characteristics, showing promise as new tools in clinical workflows. This review seeks to provide a comprehensive examination of video‐based, data‐driven models for movement disorders, including tremor, dystonia, myoclonus, chorea, tics, Parkinson's disease, and ataxia. We explore literature from 2006 to 2024 in a variety of scientific databases, with different video modalities including red‐green‐blue video of different frame rates, depth video, marker‐based approaches, multi‐perspective approaches, and multimodal video. We discover a significant trend in studies favoring pose estimation methods, with newer studies incorporating real‐time methods and end‐to‐end DL architectures, and usability is steadily increasing and rapidly approaching expert‐level performance. Likewise, we present the main limitations in the current approaches, such as limited public sources of data, lack of standardized metrics, and patient privacy. Taking inspiration from other fields, in medicine and otherwise, we propose possible future research directions including explainable artificial intelligence techniques, privacy‐preserving devices and modeling techniques, and better metric guidelines. © 2025 The Author(s). *Movement Disorders* published by Wiley Periodicals LLC on behalf of International Parkinson and Movement Disorder Society.

Movement disorders affect a substantial portion of the global population each year. Parkinson's disease (PD),^1^ for example, is a growing concern in the increasingly elderly population of many countries.^2^, ^3^, ^4^ Cerebral Palsy, a condition that can cause a combination of spasticity, dystonia, and ataxia,^5^ affects a notable percentage of births annually. Adequate phenotyping is important, as it guides etiological diagnosis and symptomatic treatment.

However, objective evaluation of movement disorders is challenging, as assessments often rely on the experience‐based judgements of clinical experts.^6^ Although objective guidelines have been developed to standardize the scoring and assessment of many disorders, interrater disagreement remains a well‐documented issue.^7^ Given their profound impact, one would expect robust, objective methods for diagnosis, severity assessments, and treatment evaluation. This is not the case. Neurophysiological tests, such as tremor registrations, can supplement clinical evaluations,^8^ but are time consuming, require special in‐hospital equipment, and an experienced rater. Furthermore, the sensitivity and specificity depend on the movement disorder phenotype and severity.^9^ Some techniques exist that rely on wearable devices, but many patients find these uncomfortable.^10^ There is a need for patient‐ and physician‐friendly tools that enable objective measurements, provide automated second opinions, and improve accessibility.^10^

Data‐driven video models, especially advanced machine learning (ML) and deep learning (DL) techniques have shown strong potential for interpreting complex data once thought beyond computational reach. Despite their potential, such methods remain uncommon in medical practice, with significant challenges hindering their integration into clinical workflows worldwide.^11^ We define data‐driven models as those that learn to associate visual input (eg, patient movement in video) with clinical outcomes by training on large datasets. Rather than following predefined rules, these models identify patterns directly from the data. To discover the direction of existing video methods, and to discuss possible future directions that could help solve the challenges facing the field, we present an overview of video‐based, data‐driven methods for assessing movement disorders.

To our knowledge, this is the most comprehensive review focused specifically on video‐based, data‐driven models for movement disorders. Six recent surveys explore related areas,^12^, ^13^, ^14^, ^15^, ^16^, ^17^ but differ in important ways: some focus on a single disorder or modality,^12^, ^13^, ^14^ while others provide broader overviews like ours.^15^, ^16^, ^17^ Our main contributions are wider coverage of different movement disorders and video approaches, an examination on why promising models remain underused in practice, and a proposal of feasible directions forward, drawing on insights from other fields. We offer a top‐down analysis of current methods, discuss their practical implications, and summarize existing applications in a way that connects technical development with clinical needs.

Our work is structured to address the following key questions about video‐based, data‐driven models: (1) what are the main approaches for designing, training, and scoring these models? (2) What strengths and weaknesses do these models have in comparison with each other? (3) What are the main challenges still limiting the application of these models in clinical practices? (4) What potential solutions could facilitate the eventual adoption of these models?

## Methods

This review follows the Preferred Reporting Items for Systematic reviews and Meta‐Analyses (PRISMA) guidelines to ensure structure and replicability for our search.^18^ We do not provide a full systematic review, as some of the points for this protocol are not applicable to all the works collected. Our target movement disorders include PD, dystonia, tremor, myoclonus, tics, chorea, and ataxia.

We focus on emerging data‐driven techniques, which are able to automatically learn from available data, and have proven to be increasingly effective to analyze video content rapidly and accurately.^19^ We examine six major scientific databases spanning medical and computational research: PubMed, ScienceDirect, SpringerLink, IEEEXplore, Web of Science, and the ACM Digital Library.

We used the following search terms: ((“computer vision” OR “machine learning” OR “pose estimation” OR “action recognition”) AND (video OR leap OR kinect OR vicon OR azure)) AND ((tremor) OR (parkinsons) OR (dystonia) OR (myoclonus) OR (chorea) OR (tics) OR (ataxia)).

For all databases, we filtered exclusively for research articles, including those published in conference proceedings. We compare our results with the included works from the identified surveys, adding articles which are relevant to our own scope.

To contextualize the studies included, and analyze their overarching trends, we first provide an overview of the evolution of these techniques from 2006 to 2024 and explain key concepts and methods for video analysis using ML. We focus on two primary ML tasks—classification and regression. Subsequently we provide an overview of the main techniques currently in use clinical practice, including the six input modalities used to encode video in modern data‐driven models, evaluating their respective strengths and weaknesses.

In addition, we examine the best approaches identified for medical video analysis. Two main strategies of data‐driven video models are explored: landmark‐based and end‐to‐end methods. These are described in terms of their methodologies, strengths, and limitations. Next, we provide a comparative discussion of video modalities and data‐driven approaches, with a focus on their limitations and key challenges, and potential future directions for clinical translation.

## Results

**FIG. 1:**
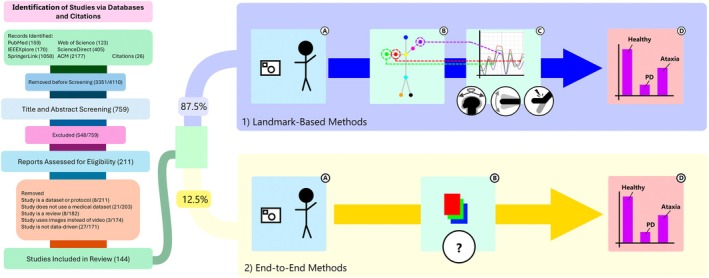
Preferred Reporting Items for Systematic reviews and Meta‐Analyses (PRISMA) flowchart summarizing the study selection process for this review, and main categorization for selected literature. The initial search yielded 4110 records from medical journals and databases. Ultimately, 144 studies (Table 1) were analyzed and summarized in this review. The two main methods used are divided in two: (1) landmark‐based methods (87.5% of results) leverage pre‐trained models like OpenPose and DeepLabCut to transform video analysis problems into time‐series analysis tasks, whereas (2) end‐to‐end methods (12.5% of results) process all information in a video at once, regardless of complexity. First, a video modality is captured (**A**). This data is fed into a video model (**B**). For landmark‐based models, features are extracted from this video model (**C**), which can be defined by an expert or created by a secondary model in the case of deep learning approaches. End‐to‐end approaches do not have this step, instead proceeding directly to the main task. The information processed by the models is used to draw a conclusion (**D**). [Color figure can be viewed at wileyonlinelibrary.com]

Our review strategy is summarized in Figure 1. Initially, we retrieved 4110 records, which included duplicates and non‐relevant results like full conference proceedings and posters. After multiple rounds of screening, we finalized a selection of 144 papers, detailed in Table 1.

**TABLE 1:** Articles surveyed using the detailed strategy.

Reference no.	Modality	Year	Disorder	Target			Tasks	Data‐driven technique	DS size	Metrics
				D	S	B				
^104^		2024	Ataxia	✓	✓		W	OP + LSTM	65/24	F1, Acc
^88^		2024	Ataxia	✓			W	OP + Transformer	143/0^d^	AUC, Acc, Spec, Rec
^150^		2024	Ataxia			✓	W	OP	95/44	KW + ANOVA
^89^		2024	Ataxia			✓	W	OP	123/0^d^	Error
^86^		2024	Dystonia	✓			Rest	OP + DeepLabCut + MoveNet	5/5	AUC
^120^		2024	Dystonia		✓	✓	F	MediaPipe + CNN	116/22	Acc, Pear
^117^		2024	Dystonia			✓	H	DeepLabCut	30/21	MWU
^78^		2024	Dystonia		✓		L	DeepLabCut + HIVE‐COTE	33/4^c^	F1, Spec, Rec, Prec
^126^		2024	PD	✓	✓		H	MediaPipe + LoR	66/24	AUC, F1
^103^		2024	PD	✓	✓		H	OP + LSTM	329/177^d^	Acc, MSE
^132^		2024	PD	✓		✓	W	VIBE + Two‐stream	32/47^b^	AUC, F1, Acc, Rec, Prec, KW
^79^		2024	PD	✓			W	HRNet + Transformers	96/95^c^	F1, Acc, Rec
^123^		2024	PD	✓			W	MediaPipe + ResNet50	30/42	Acc
^20^		2024	PD	✓			H	PIXIE + SVM	28/0^d^	AUC, F1, Acc, Rec, Prec
^130^		2024	PD	✓			H	3D CNN	40/37	AUC, Acc, Rec, Prec
^24^		2024	PD		✓		W	AlphaPose + GCN	273/0	AUC, F1, Acc, AWO
^25^		2024	PD		✓		F	3D CNN + LSTM	300/77^d^	F1, Acc, Spec, Rec, Prec
^121^		2024	Tics	✓			F	MediaPipe + Transformer	11/0^c^	AUC
^134^		2024	Tremor	✓	✓		H	AlphaPose + GCN	50/5	F1, Acc, Spec, Rec, MAE
^22^		2024	Tremor			✓	H	MediaPipe + LoR	144/10^d^	AUC, F1, bAcc, R2, Spear, MAE
^140^	RGB	2023	Ataxia	✓	✓		W	FasterRCNN + MoveNet + RF	65/24^d^	F1, Acc, MAE, Pear
^151^		2023	Ataxia	✓			W	AlphaPose + YoloV3 + GCN	50/34	F1
^77^		2023	Dystonia		✓	✓	Rest	DeepLabCut + RF	33/0^c^	MAE
^113^		2023	Dystonia			✓	W	DeepLabCut + LR	46/70	R2, MWU
^52^		2023	PD	✓	✓	✓	H, L	OP + RF	628/0	AUC, ICC, bAcc, AWO
^152^		2023	PD	✓	✓		W	VIBE + NN	40/20	AUC, F1, Acc, Prec
^91^		2023	PD	✓			W	OP + GCN	6/50^d^	F1, Acc, Spec, Rec, Prec, MR
^92^		2023	PD		✓	✓	W	OP + GCN + Two‐stream	54/26	AUC, F1, Rec, Prec, Spear, Coh
^27^		2023	PD		✓		W	3D CNN	74/0	MAE
^153^		2023	PD		✓		W	AlphaPose + OP + GCN + NN	27/0	F1, Acc, Prec, Rec
^154^		2023	PD		✓		W	MediaPipe + DT	50/0	F1, Acc, Prec, Rec
^26^		2023	PD		✓		W	MediaPipe + Gradient boosting	172/78^c^	MAE, MSE, Pear, Spear, Kendall
^106^		2023	PD		✓		H	OP + MMPose + GCN	490/0	AUC, F1, Acc, Prec, Rec, AWO
^155^		2023	PD			✓	W	OP	12/12	Pear
^93^		2023	PD	✓			H	OP + GCN	50/5	F1, Acc, Spec, Rec
^53^		2023	PD			✓	H, L	OP + GCN	1170/0	IoU, bAcc
^156^		2023	PD		✓		W	HRNet + RF	66/0	AUC, F1, Acc
^157^		2023	Tics	✓	✓		F	3D CNN	57/44	AUC, Acc, Rec, Prec
^122^		2023	Tics	✓			Rest	MediaPipe + RF + CNN	34/0^d^	AUC, F1, Acc
^139^		2023	Tremor		✓		Rest	HRNet + Transformers	61/0^d^	AUC, F1, Acc, Spec, Rec, Prec
^114^		2022	Ataxia	✓	✓		F	DeepLabCut + RR + LoR	243/58^d^	AUC, Spec, Rec, Pear
^158^		2022	Ataxia	✓			F	IntraFace + SVM + PCA	151/0	Acc, Spec, Rec
^159^		2022	Ataxia			✓	W	AlphaPose	10/5^c^	MAE, Variance
^160^		2022	Dystonia			✓	F	OpenFace + LR	93/0	Spear, R2, RMSE
^161^		2022	Dystonia			✓	F	OpenFace	185/0^d^	Spear
^95^		2022	PD	✓	✓		H	OP + MMPose + GCN	174/0	AUC, F1, Acc, AWO, Rec, Prec
^81^		2022	PD	✓			W	HRNet + GCN + Two‐stream	96/96^c^	F1, Acc, Rec
^107^		2022	PD	✓			W	OP	19/9	Acc
^108^		2022	PD	✓			H	OP + XGB	59/24	F1, Acc, Prec, Rec
^162^		2022	PD	✓			F	CNN + LoR	91/75	AUC, Acc, Spec, Rec
^49^		2022	PD	✓			H	ViTPose + Transformers	50/5	AUC, F1, Acc, Spec, Rec
^80^		2022	PD	✓			H	DeepLabCut + SVM	5/0^c^	Acc
^94^		2022	PD	✓			W	OP	68/48	AUC, Spec, Rec
^100^		2022	PD		✓		W	OP + GCN	144/0	AUC, bAcc, Prec, Rec, AWO
^28^		2022	PD		✓		H	OP + CNN	9/0	MSE
^101^		2022	PD		✓		W	OP + GCN	186/0	AUC, Acc, AWO
^48^		2022	PD		✓		W, H	VIBE + Transformers	54/0	F1, Rec, Prec
^163^		2022	PD		✓		H	PoseNet + LSTM + Two‐stream	0/12^a^	Acc, Spec, Rec
^141^		2022	PD		✓		H	MMPose + NN	779/0^d^	F1
^96^		2022	PD		✓		W	OP + RF	447/0	Acc, AWO
^124^		2022	PD		✓		H	MediaPipe + CNN	93/27^d^	Acc
^115^		2022	PD			✓	H	DeepLabCut + DT	36/11	Pear
^57^		2022	PD			✓	W	AlphaPose + OP	25/0	Spear
^164^		2022	Tics	✓			F	Two‐stream	68/0^d^	Accuracy
^105^		2021	PD	✓			W	OP + RF	20/20	Acc
^135^		2021	PD	✓			F	OpenFace + LoR	199/130	F1
^165^		2021	PD	✓			H	SVM + NN + RF	37/38^d^	Acc, Spec, Rec, MAE
^97^		2021	PD	✓			H	SSD + OP + LoR	22/20	AUC, Acc
^43^		2021	PD	✓			W	3D CNN	18/42	Acc
^42^		2021	PD	✓			W	3D CNN	11/11^d^	F2, AUC, Acc, Rec, Prec
^166^	RGB	2021	PD		✓		W	VIBE + OP + NN	50/0	MAE, MSE, Pear, Spear, Kendall
^167^		2021	PD		✓		W	AlphaPose + GCN	184/0	Acc, AWO
^168^		2021	PD		✓		W	3D CNN	35/0	F1
^169^		2021	PD		✓		H,W	OP + GCN + CNN	39/0	Acc, MCC
^44^		2021	PD		✓		H	OP + CNN + Two‐stream	157/0	Acc, AWO
^54^		2021	PD		✓		W	OP + RF	544/185	bAcc, AWO, MSE, Pear, Spear
^99^		2021	PD		✓		H	OP + SVM	55/0^d^	ICC
^170^		2021	PD		✓		W	SVM	19/0	Acc
^98^		2021	PD			✓	W	OP	63/0^d^	R, ICC
^136^		2021	Tics	✓			Rest	SVM	101/111^d^	Acc
^119^		2021	Tremor	✓			H	MediaPipe + CNN + LSTM	50/5	F1, Acc, Rec, Prec
^85^		2021	Tremor	✓			W, H	OP + LR + XGB + RF + SVM	54/24	F1, Acc, Rec, Prec
^87^		2020	Myocl.			✓	H, F	OP	10/0	Pear
^171^		2020	PD	✓	✓		H	MobileNet + SVM	20/15	AUC, Acc, Spec, Rec
^172^		2020	PD	✓			H	CNN + SVM	206/139	F1, Acc, Rec, Prec
^173^		2020	PD	✓			W	CNN + GCN	45/0	AUC, Acc, Spec, Rec
^174^		2020	PD		✓		W	SPIN + CNN	29/1	AUC, F1, Rec, Prec
^175^		2020	PD		✓		W	OP + GCN	157/0	AUC, F1, Acc, Prec, Rec, AWO
^116^		2020	PD			✓	H	DeepLabCut	39/30	Spear
^176^		2020	Ataxia	✓	✓		F	IntraFace + SVM + LoR	145/18^d^	AUC, Spec, Rec
^21^		2020	Chorea	✓			W	AlphaPose + RF + LR + NN	0/?^a^	Acc
^110^		2019	PD	✓			W	OP + NN	48/16	Acc
^177^		2019	PD	✓			H	OpenFace + K‐means	127/127	MWU
^178^		2019	PD	✓			H	CNN + LoR + NB	20/15	Acc
^179^		2019	PD		✓		H	MobileNet + SVM	60/0	Acc
^39^		2018	PD	✓	✓		L, H	CPM + RF	9/0^c^	AUC, F1, Acc, RMSE, Pear
^40^		2018	PD	✓			W	CPM + LSTM	24/0	Acc
^109^		2018	PD	✓			W	OP + DT	26/23^b^	Acc
^180^		2012	Myocl.	✓			Rest	SVM	3/0	PPV, Rec
^181^	RGB	2012	PD		✓		W	PCA + LDA	12/12	Pearson
^182^		2011	PD	✓			W	Kernel PCA	12/12	Acc
^30^		2009	PD	✓			W	PCA + LDA	7/7	Acc
^23^		2024	PD		✓		W, H	MediaPipe + LoR	31/0^c^	AUC, Acc
^58^		2024	PD			✓	W	OP	63/32	Pear
^60^		2023	PD	✓		✓	W	AlphaPose + NN	29/5	F1, Acc, Rec, Prec, Error
^59^	+RGB	2023	PD	✓			W	OP + ResNet	19/14	AUC, F1, Acc, Rec, Prec
^183^		2023	PD			✓	W	OP + NN	9/5	MAE, MSE, Pear, ICC
^62^		2022	Dystonia	✓			Rest	GCN + Multi‐stream	18/2	F1, Acc
^61^		2020	PD			✓	H	OP	5/22	Pear
^65^		2024	PD	✓			W	SVM	14/14	Acc
^184^		2024	PD		✓		H	Leap + SVM	45/0	F1, Acc, Prec, Rec
^185^		2023	PD	✓			H	Kinect + KNN	35/60	F1, Acc, Prec, Rec
^118^		2023	Tremor			✓	H	MediaPipe	0/2^a^	Error
^186^		2022	PD	✓			W	Kinect + Distance	28/14^c^	Acc
^187^		2022	PD		✓	✓	W	GCN	67/0	F1, MAE
^68^		2022	PD		✓	✓	H	SVM	48/11	Acc
^188^		2020	Ataxia		✓	✓	W	Kinect + SVM	31/20	Acc, Coh, Rec, Spec, Pear
^189^		2020	PD	✓			H	Leap + RF	16/16	Acc
^66^	RGB‐D	2019	PD	✓	✓	✓	W, L	SVM	14/12	Pear, Acc
^190^		2019	PD	✓			W	Kinect + LSTM + CNN	88/94	F1, Acc, Prec, Rec, Coh
^191^		2019	PD		✓		H	Leap + DT	21/0	Acc
^67^		2019	PD		✓		H	LR	8/0	Acc
^69^		2018	PD	✓	✓		W	BNs	30/11^d^	Acc
^192^		2018	PD	✓			H	Leap + NB	16/12^d^	AUC, Acc
^193^		2018	PD	✓			W	Kinect + RF	32/28	F1, Acc, Prec, Rec
^194^		2018	PD	✓			W	Kinect + RF	15/15	Acc
^195^		2018	PD	✓			W	KNN	28/14	Acc
^196^		2015	PD	✓			H	Kinect + SVM	8/5	Acc
^33^		2015	PD	✓			W	NNs	18/33	Acc
^197^		2015	PD		✓		H	Kinect + SVM	9/0	AUC
^71^		2024	PD	✓	✓		W	PSO + KNN + DT + SVM + LR + BN	70/30^b^	Acc, Spec, Rec, Prec
^34^		2015	PD	✓			W	RF + MR	23/26	AUC, Spec, Rec
^32^	Markers	2014	PD	✓	✓		H	SVM	13/6	Acc
^72^		2022	PD			✓	H	MediaPipe	11/0	Spear, RMSE
^73^	FPS+	2020	PD	✓			H	PoseNet + LSTM	83/94	F1, Acc, Rec, Prec
^125^		2024	Dystonia	✓			H	MediaPipe + Autoencoder	20/16^d^	Acc, Spec, Rec
^102^		2024	PD	✓			H	OP + MediaPipe + LSTM	79/34	AUC, Acc, Spec, Rec, Prec
^198^		2022	Dystonia			✓	Rest	HRNet + YOLOv3	8/0^d^	Spear, ICC
^131^		2022	PD	✓			W, G	Two‐Stream MobileNet	13/13	F1, MCC, Acc, Spec, Rec, Prec
^76^		2022	Tremor	✓			W, H	OP + RF + XGB + LoR + SVM	59/24	F1
^75^	Multiple	2021	PD	✓			ITW	Autoencoders	5/5	F1, Rec, Prec
^64^		2020	PD		✓		W	OP + LoR	14/0^d^	Acc
^199^		2019	PD	✓	✓		W, H, L	NN	16/14^d^	Acc, Spec, Rec
^200^		2018	PD	✓	✓		H	SVM	57/5	Acc

*Note*: Each block separates a different video modality, with the column Data‐driven technique showing the best performing algorithm or combination of algorithms in each study. In the Modality column, RGB methods use a single camera, whereas +RGB use two or more viewpoints and RGB‐D incorporates depth video. Tasks describe what the specified clinical tasks targeted (H: hands, W: walking, L: legs, F: face, G: gaze, ITW: in‐the‐wild, Rest: observing patient without performing a task). Target defines the goal of the study, whether it was to diagnose (D) the disorder, describe its severity (S), or to benchmark (B) a method's performance for tracking patients. “DS Size” describes the cohort used as a split of patients exhibiting the listed disorder and control patients separated by a slash (1/2 meaning a single patient with the disorder, and two control patients). For studies focusing on parkinsonian dyskinesias such as bradykinesia, parkinsonian gait, or hyperkinetic disorders primarily studied through the lenses of Parkinson's disease, the disorder is identified as “PD.” Myocl: myoclonus, Spast: spasticity. ^a^Uses synthetic data (disorder emulated by healthy individuals). ^b^Dataset used is open. ^c^Dataset is partially open. ^d^Dataset may be shared on request to the authors.

Abbreviations: (Models) OP, OpenPose; LSTM, long short‐term memory; CNN, convolutional neural network; LoR, logistic regression; SVM, support vector machine; GCN, graph convolutional neural network; RF, random forest; LR, linear regression; NN, neural network; DT, decision tree; PCA, principal component analysis; XGB, extreme gradient boosting; SSD, single‐shot detector; BN, Bayesian network; PSO, particle swarm optimization; (Metrics) F1, F1 score; F2, F2 score; AUC, receiver operating characteristic's area under the curve; Acc, accuracy; Spec, specificity; rec, recall; KW, Kruskal‐Wallis Test; Pear, Pearson correlation; Prec, precision; MSE, mean square error; AWO, accuracy‐within‐one; MAE, mean absolute error; bAcc, balanced accuracy; MWU, Mann‐Whitney *U*‐test; ICC, intra‐class coefficient; MR, miss rate; Coh, Cohen coefficient; RMSE, root mean square error.

### Video Analysis Using Data‐Driven Classification and Regression

In the works surveyed, the three most common goals for data‐driven techniques are to diagnose a movement disorder, score the disorder according to a medical scale, or obtain a kinematic measurement from a patient. These goals translate into one of the two data‐driven tasks: classification and regression, both with extensive use in literature.

In classification tasks, a model's objective is to categorize input data into predefined classes. Predicting whether a patient exhibits a tremor, for example, is a classification task. In movement disorders, classification might involve differentiating between healthy individuals and patients,^20^ or among different movement disorders.^21^

In regression tasks, the goal is to predict a continuous value.^22^ Predicting the amplitude of a tremor, for example, is a regression problem. In the context of video for movement disorders, regression is commonly applied in pose estimation, where models predict continuous coordinates representing key body positions (“landmarks”).

In some scenarios, the distinction between classification and regression blurs. For example, predicting if a patient exhibits mild or severe tremor can fit both tasks (by creating a “severe” class or by creating a threshold for the amplitude that is considered “severe”). For the task of assigning a severity level to a disorder or predicting a score from a clinical scale, some authors treat it as a classification problem,^23^, ^24^, ^25^ where others treat it as a regression problem.^26^, ^27^, ^28^

### Timeline of Data‐Driven Video Analysis for Movement Disorders

**FIG. 2:**
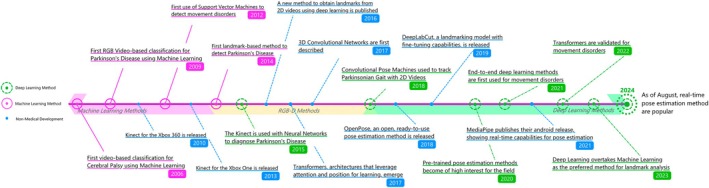
Timeline of video‐based data‐driven models for movement disorder analysis from 2006 to 2024. Recent advancements are dominated by deep learning approaches, with 64% of studies in 2023 (16/25) using these models for landmark analysis or for end‐to‐end methodologies. We identify time periods where machine learnings methods are being explored (denoted as “Machine Learning Methods”), where deep learning methods are emerging and begin gaining popularity in combination with marker‐based devices and depth sensors (denoted as “RGB‐D Methods”), and where deep learning methods using marker‐less pose estimation models gain traction (denoted as “Deep Learning Methods”).  [Color figure can be viewed at wileyonlinelibrary.com]

Model choice shapes the insights obtainable from data for either of these tasks. As the field has matured, attention has shifted toward models that leverage large, non‐medical datasets to learn patterns in human movement (see Fig. 2). Early efforts in movement disorder analysis relied on marker‐based systems,^29^ with the first data‐driven approach appearing in 2006 using silhouette classification for PD detection.^30^ These methods were similar to existing computer vision approaches for gait analysis.^31^

A shift began in 2014, marker‐based features being combined with ML models,^32^ enabling general movement analysis using computer algorithms. These methods quickly gained traction,^33^, ^34^ with pose estimation tools like the Kinect gaining popularity for capturing movement data.^35^

DL entered the field in 2015,^36^ initially through pose estimation using convolutional pose machines.^37^ These models, trained on large non‐medical datasets, reduced the need for specialized clinical data. In 2017, three‐dimensional (3D) convolutional neural networks (CNNs) introduced direct video‐based action recognition,^38^ allowing end‐to‐end clinical prediction without handcrafted features. By 2018, DL‐based landmark extraction techniques, inspired by earlier Kinect work, proved highly effective.^39^, ^40^ End‐to‐end models became practical for clinical research by 2021,^41^, ^42^, ^43^ leveraging 3D^38^ and two‐stream CNNs.^44^

Since 2022, transformer‐based models^45^ have shown strong performance in image^46^ and video tasks^47^ and were rapidly applied to medical datasets.^48^, ^49^ By 2023, traditional ML approaches reliant on pose data were largely replaced by advanced DL models better capable of handling movement data.^50^

### Video Modalities

In data‐driven workflows, designers often work with distinct, but valid data sources (“modalities”) that offer complementary perspectives on a problem. For movement disorders, multiple modalities are available, including accelerometry, electromyography, video, and patient histories. Among these, video is a topic of active discussion because of its established role in clinical practice for reviewing, sharing, and assessing patients.^51^ However, “video” encompasses a range of capture methods. Techniques like slow‐motion video offer alternative, but equally valid visual representations, each with unique advantages and limitations.

### Red‐Green‐Blue: RGB

**FIG. 3:**
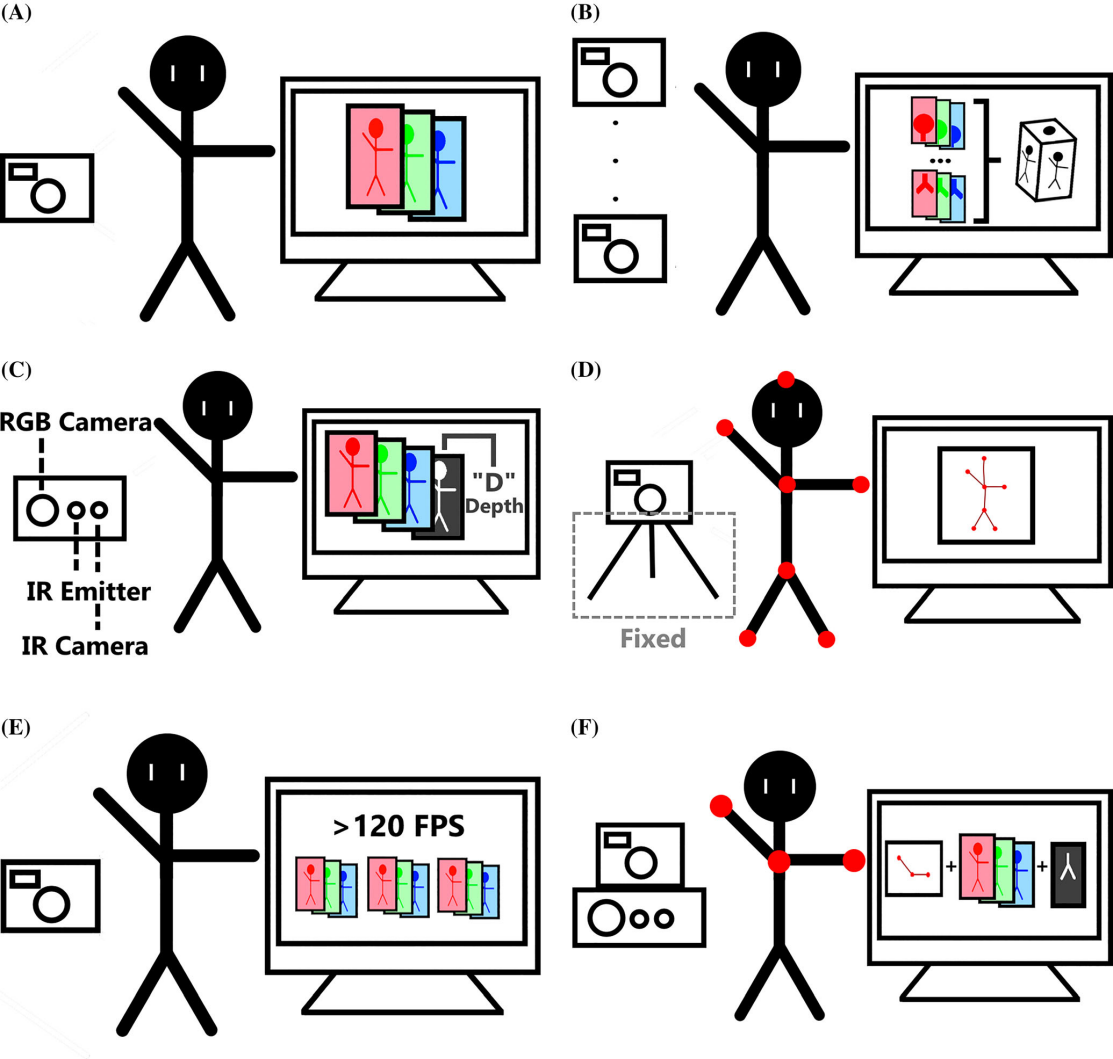
A comparison of different input modalities. The input device (left) is focused on the patient (center), sending a signal to a computer (right). (**A**) An example of a method using a red‐green‐blue (RGB) modality. A camera records video in three different channels, one for each color. (**C**) An example of a method using a Red Green Blue Depth (RGB‐D) modality. A camera equipped with a sensor capable of determining object distance (usually an infrared camera paired with an emitter) is used. (**E**) An example of a method using slow motion. A camera capable of recording at or over 120 frames per second is used, having many times the frames of a regular RGB modality. (**B**) An example of a method using a multiple RGB modality. More than one camera records different views of the patient simultaneously. A model then combines all the views into a single, shared tracking (usually in three‐dimension [3D]). (**D**) An example of a method using a marker‐based modality. The number of cameras is variable, although these must be fixed to ensure the reference system stays the same. Each frame only records the location of the markers. (**F**) An example of a method using multiple modalities. This figure combines RGB, RGB‐D, and markers, although the specific combination could be different. This approach does not require that all devices track the same view, simultaneous acquisition, nor that all inputs be a form of video. [Color figure can be viewed at wileyonlinelibrary.com]

Red‐green‐blue (RGB) video (Fig. 3A) is the standard format for full‐color video capture and the most widely used in movement disorder research (70.8% of surveyed works). It captures rich spatial and temporal detail using conventional cameras.

Diagnostic and severy classification using the Movement Disorder Society‐Unified Parkinson's Disease Rating Scale (MDS‐UPDRS) have obtained accuracies of over 80% in PD studies.^52^, ^53^, ^54^ RGB has been applied across various disorders and medical scales, as shown in Table 2. Its high dimensionality requires advanced computational methods^55^ and identifiability poses privacy concerns compared to less detailed modalities.

**TABLE 2:** A description of methods in literature by movement disorders.

Disorder	Target			Approach		Modality						Explainability		
	Diagnosis	Severity	Benchmarking	Landmarks	End‐to‐end	RGB	+RGB	RGB‐D	Markers	Slow‐mo	Multimodal	SHAP	Model‐specific	Saliency
Parkinson's disease	✓	✓	✓	✓	✓	✓	✓	✓	✓	✓	✓	✓	✓	✓
Dystonia	✓	✓	✓	✓		✓	✓				✓		✓	
Ataxia	✓	✓	✓	✓		✓						✓	✓	
Tremor	✓	✓	✓	✓		✓		✓				✓	✓	
Tics	✓	✓		✓	✓	✓							✓	
Myoclonus	✓		✓	✓	✓	✓								
Chorea			✓	✓		✓								

Elements with checkmarks indicate at least one study using that technique in this review. Model‐agnostic explainability methods and non‐RGB video modalities are mostly untested outside of Parkinson's disease.

Abbreviations: RGB, red, green, blue; +RGB, multi‐camera setups RGB; RGB‐D, red, green, blue, depth; Slow‐mo, slow motion; SHAP, Shapley additive explanations.

Current works are exploring the possibility of private data sharing between hospitals. One promising direction is federated learning, where each clinic trains models using their own data while following their own privacy protection policies. After these models are trained, they share their collective learned information with a central model without exposing patient data.^56^

### Multiple Viewpoint RGB: +RGB

Multi‐camera setups (+RGB) (Fig. 3B) capture different perspectives to reconstruct 3D representations by aligning viewpoints in a shared reference frame. This enables improved accuracy over 2D inputs,^57^ especially in assessments involving complex spatial motion (eg, gait). Separate cameras can target specific body regions (such as hands in PD^23^ and head in dystonia)^58^ for focused analysis. Studies show robust performance in challenging settings.^59^, ^60^, ^61^, ^62^

Multi‐view setups require complex calibration, high computational power, and are less suitable for telemedicine use. Most recent work favors 2D‐to‐3D deep learning alternatives. Increasing accessibility and reducing the barriers for real‐world use would heavily influence how popular this modality is for the examination of human movement. A way to do so is the implementation of automatic synchronization between different devices, with some proven applications in other fields.^63^ Current data requirements for such approaches limit their present applicability to movement disorders, but future developments could quickly change this reality.

### Depth Video: RGB‐D

Red Green Blue Depth (RGB‐D) video (Fig. 3C) combines standard color footage with depth data, capturing object distance via infrared‐based sensors. This enables real‐time^64^, ^65^ 3D tracking with low computational cost^66^ and has been used with systems like the Kinect and the Leap Motion Controller.

Some studies show performances equal to or above consumer video in PD,^67^, ^68^ but under some conditions they are overtaken by novel DL models.^69^ Performance varies by hardware and medical assessment, with strong results in gait and finger‐tracking. However, because of the construction of these devices multi‐device setups are less feasible than with other modalities^70^ and have been generally replaced by pose estimation models.

It nonetheless offers a cheap and non‐invasive alternative to other specialized sensors to acquire 3D information, while maintaining lightweight computational requirements. These systems currently rely on algorithmic, rule‐based tracking rather than data‐driven approaches, but could benefit from integration with ML techniques to improve accuracy and make them more competitive.

### Marker‐Based Modalities

Marker‐based systems use colored^32^, ^71^ or reflective^34^ markers placed on the body to simplify motion tracking (Fig. 3D). The high reliability of these devices makes them useful as gold standards, ensuring that other tracking modalities are performing as expected. These systems were critical for the first works in the field to examine movement disorders^29^ and remain a gold standard for comparing tracking methods.^71^ Their primary advantage is when traversal is involved, as they can accurately track patient locomotion regardless of environment.

Their main limitations are limited detail and usability. Markers only capture the movements they are designed to track, and they must be placed consistently in equivalent positions across patients and for specific assessments, requiring medical expertise. These markers must be physically attached to a patient, unlike other video modalities. Because of these practical obstacles, their real‐world applications are limited. They remain valuable for benchmarking, but are seldom used in recent works with the goal of diagnosing patients, as their limitations make them less suitable as alternatives to existing clinical instrumentation.

### Slow‐Motion Video

High‐frame‐rate (≥120 frames per second [FPS]) video captures fine‐grained details that can be missed by standard video recordings (Fig. 3E). Two works applied this to PD: one used a GoPro Hero7 at 180 FPS,^72^ the other an iPhone 6S at 240 FPS.^73^

These studies show promising results. Güney et al^72^ tracked tremor frequencies within ±0.174 Hz, and Lin et al^73^ achieved 77% classification accuracy using short, 15 second video clips. This modality is hypothesized to improve motion clarity when rapid movements are present by increasing temporal resolution and decreasing noise caused by low frame rates, although it has only been validated for PD (Table 2).

However, slow‐motion video generates large volumes of data and is typically unsuitable for full assessments. Models may misinterpret identical actions performed at different speeds,^74^ and direct comparisons with other modalities are lacking. More exploration is required to ensure that the advantages this modality offers are strong enough to offset the practical limitations that come with the larger file sizes and constrained video clip length of this format.

### Combined Modalities

Combined approaches integrate multiple video sources (see Fig. 3F) or fuse video with non‐video inputs like accelerometry. These systems leverage the strengths of each component while inheriting their combined limitations.

One example can be found in the work by Ye et al,^74^ which combines RGB and depth video to assess cervical dystonia, improving 3D coverage and enabling multi‐angle views. For PD, several studies report performance gains when combining data from multiple onboard sensors within a single device.^65^, ^75^, ^76^

Multimodal systems have shown superior results compared to single‐modality approaches,^75^ offering enhanced flexibility and performance in movement analysis. Separate recording and integration of optional non‐video inputs (eg, accelerometers) allow tailoring to case complexity, with more invasive modalities used only in cases where video is not enough.^76^

Despite their potential for comprehensive, machine‐assisted assessment, practical challenges hinder widespread clinical deployment. Multimodal models require consistent data across all included modalities, which complicates data collection, limits generalizability, and increases development complexity compared to single‐modality systems.

## Data‐Driven Approaches

Data‐driven approaches learn to make predictions from real‐world data, such as identifying movement disorders from video. Unlike rule‐based methods defined by experts, these models can detect complex patterns not easily described manually. As a result, they have become central to objective movement analysis. In video‐based applications, two main types are prevalent: landmark‐based models, which estimate body position and motion as an intermediate step, and end‐to‐end models, which generate predictions directly from raw video.

### Landmark‐Based Approaches

Landmark‐based methods extract key body points to simplify the tracking of kinematics relevant to movement disorder assessment (Fig. 1.1). These offer stronger privacy protections and improved interpretability compared to end‐to‐end models, as most outputs are reduced to kinematic data.^39^, ^77^, ^78^, ^79^, ^80^, ^81^ This technique is used in the majority of works reviewed.

For this method, data is first fed into a pre‐trained model (“pose estimator”), which then transforms video into coordinates compatible with simpler, less data‐hungry models. Of these pose estimators, OpenPose^82^ is the most widely adopted model for landmark‐based models. Trained on general human movement datasets,^83^, ^84^ it performs well across a variety of movement disorders.^28^, ^43^, ^52^, ^53^, ^54^, ^57^, ^59^, ^60^, ^62^, ^66^, ^76^, ^85^, ^86^, ^87^, ^88^, ^89^, ^90^, ^91^, ^92^, ^93^, ^94^, ^95^, ^96^, ^97^, ^98^, ^99^, ^100^, ^101^, ^102^, ^103^, ^104^, ^105^, ^106^, ^107^, ^108^, ^109^, ^110^ The next most popular model is DeepLabCut,^111^ which enables goal‐specific fine‐tuning, a process in which the model adjusts itself to data specific to the problem.^112^ This strategy offers tailored performance improvements, with good performance across multiple studies.^77^, ^78^, ^80^, ^86^, ^113^, ^114^, ^115^, ^116^, ^117^ Newer models like MediaPipe,^201^ designed for easy deployment on affordable devices, trade some accuracy for speed and portability, facilitating low‐cost deployment.^22^, ^23^, ^26^, ^72^, ^102^, ^118^, ^119^, ^120^, ^121^, ^122^, ^123^, ^124^, ^125^, ^126^

Once landmarks are extracted, feature engineering^127^ is used to derive interpretable metrics. Some of these features are inspired from clinical measurements, like tremor frequency,^92^ whereas others are created using a secondary model.^102^ These features are used to train a final model for the specific goal of the study. Table 1 summarizes the primary objectives: diagnosis, severity estimation, and benchmarking.

### End‐to‐End Approaches

End‐to‐end models process raw video directly into outputs like diagnosis or severity scores, bypassing the need for handcrafted features (Fig. 1.2). This approach allows automatic learning of relevant patterns, often surpassing the performance of landmark‐based methods.^128^

In the studies reviewed, two recent models have been validated using this methodology. The first method involves extending the 2D models used for image analysis.^129^ Three‐dimensional CNNs^38^ add a time dimension to handle video inputs, allowing for the interpretation of sequential data and movement patterns. These models perform well in applications like action recognition and have shown competitive results for some disorders (see Table 2).^25^, ^27^, ^42^, ^130^

Another strategy is the Multi‐Stream CNN,^44^ which processes different input types through separate branches. This setup supports hybrid pipelines, which combines pose data with raw video,^131^ enabling flexible integration of multiple data types.^58^, ^92^, ^132^

## Discussion

The literature reveals a diverse range of techniques, each with strengths and limitations shaped by the needs of patients, the nature of the movement disorder, and its clinical context. Recent work increasingly adopts DL models adapted from other domains. Marker‐less color video dominates, with growing interest in data‐driven methods, particularly those using 2D pose estimation.

Despite recent advances, explainability and replicability remain key challenges to building trust in these models among clinicians and patients. In addition, patient privacy is an important issue to be solved before implementation in clinical use.

Video modalities determine what factors constrain practical use in medicine. Most current research favors accessible, marker‐less RGB methods that reduce equipment needs and increase patient comfort. This method has now reached performance levels similar to other capture modalities, although some challenges still remain.

It is important to note that a majority of available modalities are mainly focused on PD and remain unexplored for other movement disorders. Multi‐camera and multimodal setups have been tested in dystonia, and depth video has shown promise for tremor, but broader validation efforts are lacking. For the future, increased temporal resolution of slow‐motion video and tracking of multiple body parts could benefit diagnosis for a variety of movement disorders.

Marker‐based systems remain the accuracy gold standard, but are often impractical in clinical settings because of accessibility issues. Enhanced modalities like +RGB and slow‐motion video offer improved detail and perspective while retaining RGB's accessibility. +RGB adds multi‐angle views and coverage of more body parts, whereas slow‐motion improves clarity for fast movements and reduces artifacts like motion blur. Still, practical limitations, such as processing demands and data size, hinder routine use. Advances in multi‐view integration and lightweight models are needed to make these methods more viable.

Combining modalities is an area currently being explored both in medicine and in computer science. Models with the capacity to include information from different data sources can study movement disorders more thoroughly, for example, video may suffer from loss of fine detail during rapid movements, but by combining it with accelerometry one can benefit from the strengths of both. These approaches can be tailored to the specific needs of a patient and to the difficulty of each case. For a realistic implementation with currently available models, however, there are significant data constraints as every modality requires its own set of data for each patient.

Data‐driven approaches enable the diagnosis and monitoring of movement disorders in an automated, objective manner. Depending on the specific approach used, it is possible to balance performance, interpretability, and resource use. Unlike traditional methods requiring explicit feature definitions, these models automatically extract movement features.

Landmark‐based models, such as OpenPose,^82^ DeepLabCut,^202^ and MediaPipe,^201^ provide robust 2D and 3D tracking of body parts with real‐time and high‐accuracy options using video already obtained during neurological examinations. They are effective across various movement disorders, with OpenPose widely applied in all movement disorders included in this review (see Table 2, “Landmarks”). Challenges remain in optimizing feature extraction from body poses.

End‐to‐end models, like 3D CNNs and Multi‐Stream CNNs, have so far been validated with PD and tics, but have been limited by small medical datasets. Developing new strategies to learn more effectively from limited datasets is critical for future use with movement disorders, where data is scarce.

Explainability refers to the ability of a model to provide a transparent and interpretable reasoning behind its outputs, enabling clinicians to understand and trust automated decisions. In most cases, models return only a prediction without a means to assess the reasoning behind it, an issue often referred to as the “black box” problem.

Tools that can automatically make decisions may cause harm when improperly deployed, shown through hard learned lessons in the history of software in medicine.^133^ The nature of medical work is that this harm befalls on the patient, making automated tools that can make silent mistakes unacceptable. For movement disorders, a misdiagnosis may cause significant and long‐lasting impacts in the patient's quality of life. A patient with myoclonus misdiagnosed with essential tremor, for example, may not undergo studies to determine the underlying etiology and lead to an improper treatment. Black box models, then, are unsuited for use in clinical work.

To address this, explainability approaches aim to make model decisions more transparent, thereby improving trust in and understanding of automated solutions. Currently, two types of strategies have been tested in our reviewed literature: model‐specific, and model‐independent.

Model‐specific methods enhance explainability by leveraging a model's learning process. For instance, vision transformers^46^ are able to show the model's “attention,” ^49,79,122,134^ whereas decision tree models assess feature importance.^32^, ^76^, ^85^, ^92^, ^96^, ^114^, ^122^, ^131^, ^135^, ^136^ The latter approach has been tested for PD, dystonia, ataxia, tics, and tremor examinations (see Table 2) to identify critical movement features. Decision‐tree based approaches have shown that combining hand and gait assessments enhances PD model performance,^23^ whereas attention has validated that models successfully focus on medical‐relevant features such as finger acceleration for ataxia diagnosis in finger‐tapping tests.^114^ By examining whether these features are in line with what would be expected of a movement disorder, it is possible to determine if a model is making decisions based on relevant medical information.

Although powerful, model‐specific explainability methods are not compatible with many novel models, such as 3D CNNs. In these cases, it may be preferable to use model‐independent explainability techniques which are independent from other modelling decisions. Techniques such as saliency maps^137^ can provide visual explanations by highlighting input regions most relevant to predictions.^25^, ^41^, ^60^, ^130^, ^132^ With this technique, it is possible to visually inspect whether a model's decision‐making process is using areas of the video relevant to a diagnosis. In PD, it has been shown how models learn to focus on the thumb and tips of the fingers even when no features are explicitly defined.^130^

There are also advantages beyond trust that can be gained by implementing transparency measures into data‐driven models. Techniques such as Shapley additive explanations (SHAP)^138^ have been used to suggest that right hand movements in PD provide stronger features for models, compared to the equivalent movements in the left hand,^103^ and have proven effective in other single‐disorder studies of PD and ataxia.^23^, ^54^, ^60^, ^122^, ^139^, ^140^ These insights may aid in the development of more accurate medical tests, or contribute to the discovery of new biomarkers.

Despite the importance of explainability methods for the creation of trustworthy data‐driven solutions, only a small percentage of works (21%, 30/144 works) consider explainability during model evaluation, and those that do are clustered on a small number of movement disorders and modalities (see Table 2).

Replicability is a critical aspect of data‐driven models. Since the only conclusions that can be drawn about model performance are inherently statistical, comparing algorithms trained and evaluated in the same way is necessary. With a disagreement on what approach to use (classification or regression), inconsistent use of metrics, and private data, it is almost impossible to determine which models are best in the current literature.

For example, consider two studies on PD severity using the MDS‐UPDRS scale. One study frames this as a classification problem,^141^ whereas the other treats it as a regression problem.^27^ Despite targeting the same movement disorder and severity scale, these different formulations have different metrics, design considerations, and models that cannot be compared.^142^

Metric choice can also impact whether works are compatible with each other. In a finger tapping test for PD, for example, a model might be judged by how closely it matches a clinician's score for severity in MDS‐UPDRS. One approach is to use accuracy,^141^ which assumes an exact reference score. Because multiple clinicians may disagree on a specific score, another sensible approach is to treat the score as an approximation to the real severity of the observed movement disorder and use metrics that allow for a margin of error. An example of this is accuracy‐within‐one (AWO),^43^ a metric that counts scores within one level of the reference as correct. Neither of these assumptions is inherently better, but if studies do not report overlapping metrics it is not possible to determine which model is best.^142^

Standardizing evaluation is a critical step to advancing the field. A solution being implemented in other areas of data‐driven modelling for medicine is “problem fingerprinting,”^143^ which suggests choosing metrics based on the specifics of the problem rather than on the properties of a model. For movement disorders, this is particularly important for class imbalance and prevalence, because some disorders are more common than others. Metrics like accuracy may misrepresent a model's performance by assuming that all classes are distributed equally within the study's population.

Even when studies share in their design decisions, any evaluations lack generalizability without shared datasets. Among 144 studies surveyed, none consistently used the same dataset, largely because of a lack of available shared data. Of existing open datasets, only one has been consistently used by multiple studies,^49^, ^93^, ^119^, ^134^ TIM‐TREMOR,^144^ but this is no longer available.

The scarcity of shared data hinders evaluating of whether models can effectively differentiate between movement disorders. Most studies focus on a single disorder, typically PD (see Table 2). Although valuable, these results leave a critical question unanswered: can models distinguish between different movement disorders?

Addressing data scarcity in particular is challenging. A large, general benchmarking dataset is ideal, but currently impractical. Privacy‐preserving modelling and data encoding, however, may be feasible for increasing model replicability. For pose estimation, kinematic data can be extracted using multiple models (as seen in partially open data, Table 1). For end‐to‐end methods, techniques like face morphing or autoencoders show promise for anonymizing videos while retaining utility.

The current landscape is shifting rapidly, supported by tools like OpenPose and advances in deep learning, including neural networks and transformers. These models are likely to shape future developments in movement disorder analysis.

There are many sources for inspiration in how to continue to improve and push these techniques forward. For example, novel vision language models can be adapted as video analysis tools, leveraging large text datasets to incorporate data‐driven descriptions of patient movements in a format easily understandable by non‐specialists.^145^ Video may also not be the only possible approach for non‐invasive patient measurements. Advances in radar technology,^146^ Wi‐Fi–based action recognition,^147^, ^148^ and neuromorphic (“brain‐like”) cameras that record events instead of complete images^149^ suggest that non‐intrusive and private movement analysis could soon be possible, facilitating data sharing to build more powerful clinical models.

## Conclusion

Although challenges remain, the future is promising. Objective, data‐driven measurements based on advanced DL models will soon enable clinicians to address pressing issues by expanding telemedicine, enhancing specialized care, enabling non‐invasive patient monitoring, and developing patient‐centered diagnostic tools.

With different modalities and techniques available depending on the specific movement disorder, clinical setting, and patient needs, it is now more feasible than ever to have multiple non‐invasive methods to examine patients objectively and accurately. For future works, studies are required to show if these results will generalize to new hospitals, populations, and disorders.

## Author Roles

(1) Research Project: A. Design, B. Execution, C. Analysis; (2) Statistical Analysis: A. Design, B: Execution, C. Review and Critique; (3) Manuscript Preparation: A. Writing of the First Draft, B. Review and Critique.

R.M.‐G.‐P.: 1A, 1B, 1C, 2D, 2B, 2C, 3A.

L.H.K.: 1B, 1C, 2C, 3B.

G.A.: 1A, 1B, 1C, 2C, 3B.

M.A.J.T.: 1A, 1B, 1C, 2C, 3B.

## Financial Disclosures

The authors declare that financial support was received for the authorship of this review (NWO Grant File 20429). Disclosure of all funding since last year: all authors are employed as stated in affiliations. R.M.G.P. and L.H.K. report no other sources of funding. G.A. reports a grant from CogniGron. M.A.J.T. reports grants from the Netherlands Organization for Health Research and Development ZonMW Topsubsidie (91218013), the European Fund for Regional Development from the European Union (01492947), the province of Friesland, the Dystonia Medical Research Foundation, and the Dystonie Wetenschapsfonds and unrestricted grants from Actelion and Merz.

## Data Availability

Data sharing is not applicable to this article as no new data were created or analyzed in this study.

## References

[1] Armstrong MJ , Okun MS . Diagnosis and treatment of Parkinson disease: a review. JAMA 2020;323(6):548–560. 10.1001/jama.2019.22360 32044947

[2] Singhal BS , Khadilkar SV . Chapter 114 ‐ neurology in the developing world. In: Biller J , Ferro JM , eds. Handbook of Clinical Neurology. Neurologic Aspects of Systemic Disease Part III. Vol. 121. Amsterdam: Elsevier; 2014:1773–1782. 10.1016/B978-0-7020-4088-7.00114-0. 24365446

[3] Qi S , Yin P , Wang L , et al. Prevalence of Parkinson's disease: a community‐based study in China. Mov Disord 2021;36(12):2940–2944. 10.1002/mds.28762 34390510

[4] Marras C , Beck JC , Bower JH , et al. Prevalence of Parkinson's disease across North America. Npj Park Dis 2018;4(1):1–7. 10.1038/s41531-018-0058-0 PMC6039505 30003140

[5] Vitrikas K , Dalton H , Breish D . Cerebral Palsy: An Overview. Am Fam Physician 2020;101(4):213–220. 32053326

[6] Goetz CG , Tilley BC , Shaftman SR , et al. Movement Disorder Society‐sponsored revision of the unified Parkinson's disease rating scale (MDS‐UPDRS): scale presentation and clinimetric testing results. Mov Disord 2008;23(15):2129–2170. 10.1002/mds.22340 19025984

[7] Eggink H , Kremer D , Brouwer OF , et al. Spasticity, dyskinesia and ataxia in cerebral palsy: are we sure we can differentiate them? Eur J Paediatr Neurol EJPN 2017;21(5):703–706. 10.1016/j.ejpn.2017.04.1333 28549726

[8] Kassavetis P , Chen R , Ganos C , et al. Global perceptions and utilization of clinical neurophysiology in movement disorders. Mov Disord Clin Pract 2024;11(4):346–351. 10.1002/mdc3.13974 38341649 PMC10982593

[9] van der Veen S , Klamer MR , Elting JWJ , Koelman JHTM , van der Stouwe AMM , et al. The diagnostic value of clinical neurophysiology in hyperkinetic movement disorders: a systematic review. Parkinsonism Relat Disord 2021;89:176–185. 10.1016/j.parkreldis.2021.07.033 34362669

[10] Cubo E , Delgado‐López PD . Telemedicine in the Management of Parkinson's disease: achievements, challenges, and future perspectives. Brain Sci 2022;12(12):1735. 10.3390/brainsci12121735 36552194 PMC9775481

[11] Saraswat D , Bhattacharya P , Verma A , et al. Explainable AI for healthcare 5.0: opportunities and challenges. IEEE Access 2022;10:84486–84517. 10.1109/ACCESS.2022.3197671

[12] Park KW , Mirian MS , McKeown MJ . Artificial intelligence‐based video monitoring of movement disorders in the elderly: a review on current and future landscapes. Singapore Med J 2024;65(3):141–149. 10.4103/singaporemedj.SMJ-2023-189 38527298 PMC11060643

[13] Amo‐Salas J , Olivares‐Gil A , García‐Bustillo Á , García‐García D , Arnaiz‐González Á , Cubo E . Computer vision for Parkinson's disease evaluation: a survey on finger tapping. Health 2024;12(4):439. 10.3390/healthcare12040439 PMC10888014 38391815

[14] Amprimo G , Masi G , Olmo G , Ferraris C . Deep learning for hand tracking in Parkinson's disease video‐based assessment: current and future perspectives. Artif Intell Med 2024;154:102914. 10.1016/j.artmed.2024.102914 38909431

[15] Tang W , van Ooijen PMA , Sival DA , Maurits NM . Automatic two‐dimensional & three‐dimensional video analysis with deep learning for movement disorders: a systematic review. Artif Intell Med 2024;156:102952. 10.1016/j.artmed.2024.102952 39180925

[16] Friedrich MU , Relton S , Wong D , Alty J . Computer vision in clinical neurology: a review. JAMA Neurol 2025;82(4):407–415. 10.1001/jamaneurol.2024.5326 39960732

[17] Wong DC , Williams S . Artificial intelligence analysis of videos to augment clinical assessment: an overview. Neural Regen Res 2024;19(4):717. 10.4103/1673-5374.382249 37843200 PMC10664118

[18] Page MJ , Moher D , Bossuyt PM , et al. PRISMA 2020 explanation and elaboration: updated guidance and exemplars for reporting systematic reviews. BMJ 2021;372:n160. 10.1136/bmj.n160 33781993 PMC8005925

[19] Zhu Y , Li X , Liu C , et al. A Comprehensive Study of Deep Video Action Recognition; Published online December 2020. 10.48550/arXiv.2012.06567.

[20] Mifsud J , Embry KR , Macaluso R , et al. Detecting the symptoms of Parkinson's disease with non‐standard video. J NeuroEngineering Rehabil 2024;21:72. 10.1186/s12984-024-01362-5 PMC11067123 38702705

[21] Goyal D , Rao Jerripothula K , Mittal A . Detection of gait abnormalities caused by neurological disorders. 2020 IEEE 22nd International Workshop on Multimedia Signal Processing (MMSP). Piscataway, NJ: IEEE; 2020:1–6. 10.1109/MMSP48831.2020.9287163.

[22] Friedrich MU , Roenn AJ , Palmisano C , et al. Validation and application of computer vision algorithms for video‐based tremor analysis. Npj Digit Med 2024;7(1):1–13. 10.1038/s41746-024-01153-1 38906946 PMC11192937

[23] Deng D , Ostrem JL , Nguyen V , et al. Interpretable video‐based tracking and quantification of parkinsonism clinical motor states. Npj Park Dis 2024;10(1):122. 10.1038/s41531-024-00742-x PMC11199701 38918385

[24] Guo R , Xie Z , Zhang C , Qian X . Causality‐enhanced multiple instance learning with graph convolutional networks for parkinsonian freezing‐of‐gait assessment. IEEE Trans Image Process 2024;33:3991–4001. 10.1109/TIP.2024.3416052 38913508

[25] Huang J , Lin L , Yu F , et al. Parkinson's severity diagnosis explainable model based on 3D multi‐head attention residual network. Comput Biol Med 2024;170:107959. 10.1016/j.compbiomed.2024.107959 38215619

[26] Islam MS , Rahman W , Abdelkader A , et al. Using AI to measure Parkinson's disease severity at home. NPJ Digit Med 2023;6:156. 10.1038/s41746-023-00905-9 37608206 PMC10444879

[27] Eguchi K , Takigawa I , Shirai S , et al. Gait video‐based prediction of unified Parkinson's disease rating scale score: a retrospective study. BMC Neurol 2023;23:358. 10.1186/s12883-023-03385-2 37798685 PMC10552271

[28] Theofilou PA , Tsatiris G , Kollias S . Automatic assessment of Parkinson's patients' dyskinesia using non‐invasive machine learning methods. 2022 International Conference on Interactive Media, Smart Systems and Emerging Technologies (IMET). Piscataway, NJ: IEEE; 2022:1–4. 10.1109/IMET54801.2022.9929825.

[29] Meinecke L , Breitbach‐Faller N , Bartz C , Damen R , Rau G , Disselhorst‐Klug C . Movement analysis in the early detection of newborns at risk for developing spasticity due to infantile cerebral palsy. Hum Mov Sci 2006;25(2):125–144. 10.1016/j.humov.2005.09.012 16458381

[30] Cho CW , Chao WH , Lin SH , Chen YY . A vision‐based analysis system for gait recognition in patients with Parkinson's disease. Expert Syst Appl 2009;36(3, Part 2):7033–7039. 10.1016/j.eswa.2008.08.076

[31] Boulgouris NV , Chi ZX . Gait recognition using radon transform and linear discriminant analysis. IEEE Trans Image Process 2007;16(3):731–740. 10.1109/TIP.2007.891157 17357733

[32] Khan T , Nyholm D , Westin J , Dougherty M . A computer vision framework for finger‐tapping evaluation in Parkinson's disease. Artif Intell Med 2014;60(1):27–40. 10.1016/j.artmed.2013.11.004 24332155

[33] Ťupa O , Procházka A , Vyšata O , et al. Motion tracking and gait feature estimation for recognising Parkinson's disease using MS Kinect. Biomed Eng Online 2015;14(1):97. 10.1186/s12938-015-0092-7 26499251 PMC4619468

[34] Wahid F , Begg RK , Hass CJ , Halgamuge S , Ackland DC . Classification of Parkinson's disease gait using spatial‐temporal gait features. IEEE J Biomed Health Inform 2015;19(6):1794–1802. 10.1109/JBHI.2015.2450232 26551989

[35] Han J , Shao L , Xu D , Shotton J . Enhanced computer vision with Microsoft Kinect sensor: a review. IEEE Trans Cybern 2013;43(5):1318–1334. 10.1109/TCYB.2013.2265378 23807480

[36] LeCun Y , Bengio Y , Hinton G . Deep learning. Nature 2015;521(7553):436–444. 10.1038/nature14539 26017442

[37] Wei SE , Ramakrishna V , Kanade T , Sheikh Y . Convolutional pose machines. IEEE Computer Society. Piscataway, NJ: IEEE; 2016:4724–4732. 10.1109/CVPR.2016.511.

[38] Tran D , Wang H , Torresani L , Ray J , LeCun Y , Paluri M . A Closer Look at Spatiotemporal Convolutions for Action Recognition; Published online April 2018. 10.48550/arXiv.1711.11248.

[39] Li MH , Mestre TA , Fox SH , Taati B . Vision‐based assessment of parkinsonism and levodopa‐induced dyskinesia with pose estimation. J NeuroEngineering Rehabil 2018;15:97. 10.1186/s12984-018-0446-z PMC6219082 30400914

[40] Li MH , Mestre TA , Fox SH , Taati B . Automated assessment of levodopa‐induced dyskinesia: evaluating the responsiveness of video‐based features. Parkinsonism Relat Disord 2018;53:42–45. 10.1016/j.parkreldis.2018.04.036 29748112

[41] Guayacán LC , Martínez F . Visualising and quantifying relevant parkinsonian gait patterns using 3D convolutional network. J Biomed Inform 2021;123:103935. 10.1016/j.jbi.2021.103935 34699990

[42] Cao X , Xue Y , Chen J , et al. Video based shuffling step detection for parkinsonian patients using 3D convolution. IEEE Trans Neural Syst Rehabil Eng 2021;29:641–649. 10.1109/TNSRE.2021.3062416 33635791

[43] Li H , Shao X , Zhang C , Qian X . Automated assessment of parkinsonian finger‐tapping tests through a vision‐based fine‐grained classification model. Neurocomputing 2021;441:260–271. 10.1016/j.neucom.2021.02.011

[44] Simonyan K , Zisserman A . Two‐Stream Convolutional Networks for Action Recognition in Videos; Published online November 2014. 10.48550/arXiv.1406.2199.

[45] Brown TB , Mann B , Ryder N , et al. Language models are few‐shot learners; Published online July 2020. 10.48550/arXiv.2005.14165.

[46] Dosovitskiy A , Beyer L , Kolesnikov A , et al. An image is worth 16x16 words: transformers for image recognition at scale; Published online June 2021. 10.48550/arXiv.2010.11929.

[47] Bertasius G , Wang H , Torresani L . Is space‐time attention all you need for video understanding? Published online June 2021. 10.48550/arXiv.2102.05095.

[48] Endo M , Poston KL , Sullivan EV , Fei‐Fei L , Pohl KM , Adeli E . GaitForeMer: Self‐Supervised Pre‐Training of Transformers Via Human Motion Forecasting for Few‐Shot Gait Impairment Severity Estimation. Med Image Comput Comput‐Assist Interv MICCAI Int Conf Med Image Comput Comput‐Assist Interv. Cham, Switzerland: Springer; Vol. 13438; 2022:130–139. 10.1007/978-3-031-16452-1_13. PMC9635991 36342887

[49] Zhang M , Zhao N , Yu Y , et al. A simple yet effective hand pose tremor classification algorithm to diagnosis Parkinsons disease. 2022 IEEE International Conference on Bioinformatics and Biomedicine (BIBM). Piscataway, NJ: IEEE; 2022:887–890. 10.1109/BIBM55620.2022.9995709.

[50] Zhang S , Tong H , Xu J , Maciejewski R . Graph convolutional networks: a comprehensive review. Comput Soc Netw 2019;6(1):11. 10.1186/s40649-019-0069-y 37915858 PMC10615927

[51] Sambati L , Baldelli L , Calandra Buonaura G , et al. Observing movement disorders: best practice proposal in the use of video recording in clinical practice. Neurol Sci 2019;40(2):333–338. 10.1007/s10072-018-3639-0 30448965

[52] Morinan G , Dushin Y , Sarapata G , et al. Computer vision quantification of whole‐body parkinsonian bradykinesia using a large multi‐site population. Npj Park Dis. 2023;9(1):1–12. 10.1038/s41531-023-00454-8 PMC9883391 36707523

[53] Sarapata G , Dushin Y , Morinan G , et al. Video‐based activity recognition for automated motor assessment of Parkinson's disease. IEEE J Biomed Health Inform 2023;27(10):5032–5041. 10.1109/JBHI.2023.3298530 37490373

[54] Rupprechter S , Morinan G , Peng Y , et al. A clinically interpretable computer‐vision based method for quantifying gait in Parkinson's disease. Sensors 2021;21(16):5437. 10.3390/s21165437 34450879 PMC8399017

[55] Chai J , Zeng H , Li A , Ngai EWT . Deep learning in computer vision: a critical review of emerging techniques and application scenarios. Mach Learn Appl 2021;6:100134. 10.1016/j.mlwa.2021.100134

[56] Sheller MJ , Edwards B , Reina GA , et al. Federated learning in medicine: facilitating multi‐institutional collaborations without sharing patient data. Sci Rep 2020;10(1):12598. 10.1038/s41598-020-69250-1 32724046 PMC7387485

[57] Sabo A , Gorodetsky C , Fasano A , Iaboni A , Taati B . Concurrent validity of Zeno instrumented walkway and video‐based gait features in adults with Parkinson's disease. IEEE J Transl Eng Health Med 2022;10:1–11. 10.1109/JTEHM.2022.3180231 PMC9252334 35795874

[58] Hou JC , Thonnat M , Bartolomei F , McGonigal A . Automated video analysis of emotion and dystonia in epileptic seizures. Epilepsy Res 2022;184:106953. 10.1016/j.eplepsyres.2022.106953 35753205

[59] Stenum J , Hsu MM , Pantelyat AY , Roemmich RT . Clinical gait analysis using video‐based pose estimation: multiple perspectives, clinical populations, and measuring change. PLOS Digit Health 2024;3(3):e0000467. 10.1371/journal.pdig.0000467 38530801 PMC10965062

[60] Kaur R , Motl RW , Sowers R , Hernandez ME . A vision‐based framework for predicting multiple sclerosis and Parkinson's disease gait dysfunctions—a deep learning approach. IEEE J Biomed Health Inform 2023;27(1):190–201. 10.1109/JBHI.2022.3208077 36126031

[61] Gholami M , Ward R , Mahal R , et al. Automatic labeling of Parkinson's disease gait videos with weak supervision. Med Image Anal 2023;89:102871. 10.1016/j.media.2023.102871 37480795

[62] Pang Y , Christenson J , Jiang F , et al. Automatic detection and quantification of hand movements toward development of an objective assessment of tremor and bradykinesia in Parkinson's disease. J Neurosci Methods 2020;333:108576. 10.1016/j.jneumeth.2019.108576 31923452

[63] Wang C , Reza MA , Vats V , et al. Deep learning‐based 3D reconstruction from multiple images: a survey. Neurocomputing. 2024;597:128018. 10.1016/j.neucom.2024.128018

[64] Ferraris C , Votta V , Nerino R , Chimienti A , Priano L , Mauro A . At‐home assessment of postural stability in parkinson's disease: a vision‐based approach. J Ambient Intell Humaniz Comput 2024;15(5):2765–2778. 10.1007/s12652-023-04553-5

[65] Ferraris C , Nerino R , Chimienti A , et al. Feasibility of home‐based automated assessment of postural instability and lower limb impairments in Parkinson's disease. Sensors (Basel) 2019;19(5):1129. 10.3390/s19051129 30841656 PMC6427119

[66] Sabo A , Mehdizadeh S , Ng KD , Iaboni A , Taati B . Assessment of parkinsonian gait in older adults with dementia via human pose tracking in video data. J NeuroEngineering Rehabil 2020;17(1):97. 10.1186/s12984-020-00728-9 PMC7362631 32664973

[67] Lee WL , Sinclair NC , Jones M , et al. Objective evaluation of bradykinesia in Parkinson's disease using an inexpensive marker‐less motion tracking system. Physiol Meas 2019;40(1):014004. 10.1088/1361-6579/aafef2 30650391

[68] Guo Z , Zeng W , Yu T , et al. Vision‐based finger tapping test in patients with Parkinson's disease via spatial‐temporal 3D hand pose estimation. IEEE J Biomed Health Inform 2022;26(8):3848–3859. 10.1109/JBHI.2022.3162386 35349459

[69] Dranca L , de Abetxuko Ruiz de Mendarozketa L , Goñi A , et al. Using Kinect to classify Parkinson's disease stages related to severity of gait impairment. BMC Bioinformatics 2018;19:471. 10.1186/s12859-018-2488-4 30526473 PMC6288944

[70] Tölgyessy M , Dekan M , Chovanec Ľ , Hubinský P . Evaluation of the azure Kinect and its comparison to Kinect V1 and Kinect V2. Sensors 2021;21(2):413. 10.3390/s21020413 33430149 PMC7827245

[71] Kour N , Gupta S , Arora S . A vision‐based hybrid ensemble learning approach for classification of gait disorders. Multimed Tools Appl 2024;84:17597–17644. 10.1007/s11042-024-19673-z

[72] Güney G , Jansen TS , Dill S , et al. Video‐based hand movement analysis of Parkinson patients before and after medication using high‐frame‐rate videos and MediaPipe. Sensors 2022;22(20):7992. 10.3390/s22207992 36298342 PMC9611677

[73] Lin B , Luo W , Luo Z , et al. Bradykinesia recognition in Parkinson's disease via single RGB video. ACM Trans Knowl Discov Data 2020;14(2):1–19. 10.1145/3369438

[74] Sun W , Su R , Yu Q , Xu D . Slow motion matters: a slow motion enhanced network for weakly supervised temporal action localization. IEEE Trans Circuits Syst Video Technol 2023;33(1):354–366. 10.1109/TCSVT.2022.3201540

[75] Heidarivincheh F , McConville R , Morgan C , et al. Multimodal classification of Parkinson's disease in home environments with resiliency to missing modalities. Sensors 2021;21(12):4133. 10.3390/s21124133 34208690 PMC8235443

[76] Kovalenko E , Shcherbak A , Somov A , et al. Detecting the Parkinson's disease through the simultaneous analysis of data from wearable sensors and video. IEEE Sens J 2022;22(16):16430–16439. 10.1109/JSEN.2022.3191864

[77] Haberfehlner H , van de Ven SS , van der Burg SA , et al. Towards automated video‐based assessment of dystonia in dyskinetic cerebral palsy: a novel approach using markerless motion tracking and machine learning. Front Robot AI 2023;10. 10.3389/frobt.2023.1108114 PMC10018017 36936408

[78] Haberfehlner H , Roth Z , Vanmechelen I , et al. A novel video‐based methodology for automated classification of dystonia and choreoathetosis in dyskinetic cerebral palsy during a lower extremity task. Neurorehabil Neural Repair 2024;38(7):479–492. 10.1177/15459683241257522 38842031

[79] Ma L , Huo H , Liu W , Zhao C , Wang J , Xu N . Twin‐tower transformer network for skeleton‐based Parkinson's disease early detection. Complex Intell Syst 2024;10:6745. 10.1007/s40747-024-01507-y

[80] Baker S , Tekriwal A , Felsen G , et al. Automatic extraction of upper‐limb kinematic activity using deep learning‐based markerless tracking during deep brain stimulation implantation for Parkinson's disease: a proof of concept study. PLoS One 2022;17(10):e0275490. 10.1371/journal.pone.0275490 36264986 PMC9584454

[81] He Y , Yang T , Yang C , Zhou H . Integrated equipment for Parkinson's disease early detection using graph convolution network. Electronics 2022;11(7):1154. 10.3390/electronics11071154

[82] Cao Z , Hidalgo G , Simon T , Wei SE , Sheikh Y . OpenPose: Realtime multi‐person 2D pose estimation using part affinity fields; Published online May 2019. 10.48550/arXiv.1812.08008. 31331883

[83] Lin TY , Maire M , Belongie S , et al. Microsoft COCO: common objects in context; Published online February 2015 10.48550/arXiv.1405.0312.

[84] Andriluka M , Pishchulin L , Gehler P , Schiele B . 2D human pose estimation: new benchmark and state of the art analysis. 2014 IEEE Conference on Computer Vision and Pattern Recognition. Piscataway, NJ: IEEE; 2014:3686–3693. 10.1109/CVPR.2014.471.

[85] Kovalenko E , Talitckii A , Anikina A , et al. Distinguishing between Parkinson's disease and essential tremor through video analytics using machine learning: a pilot study. IEEE Sens J 2021;21(10):11916–11925. 10.1109/JSEN.2020.3035240

[86] Shen X , Hui Z , Zhang B , Liu J , Ma L . MADS: A Deep Learning‐Based Toolkit for Motion Analysis in Movement Disorders. Proceedings of the 2023 4th International Symposium on Artificial Intelligence for Medicine Science. ISAIMS ‘23. Association for Computing Machinery. New York: Association for Computing Machinery; 2024:840–845. 10.1145/3644116.3644258.

[87] Hyppönen J , Hakala A , Annala K , et al. Automatic assessment of the myoclonus severity from videos recorded according to standardized unified myoclonus rating scale protocol and using human pose and body movement analysis. Seizure 2020;76:72–78. 10.1016/j.seizure.2020.01.014 32035366

[88] Eguchi K , Yaguchi H , Uwatoko H , et al. Feasibility of differentiating gait in Parkinson's disease and spinocerebellar degeneration using a pose estimation algorithm in two‐dimensional video. J Neurol Sci 2024;464:123158. 10.1016/j.jns.2024.123158 39096835

[89] L'Italien GJ , Oikonomou EK , Khera R , et al. Video‐based kinematic analysis of movement quality in a phase 3 clinical trial of Troriluzole in adults with spinocerebellar ataxia: a post hoc analysis. Neurol Ther 2024;13(4):1287–1301. 10.1007/s40120-024-00625-6 38814532 PMC11263303

[90] Morgan C , Tonkin EL , Masullo A , et al. A multimodal dataset of real world mobility activities in Parkinson's disease. Sci Data 2023;10(1):918. 10.1038/s41597-023-02663-5 38123584 PMC10733419

[91] Zhang J , Lim J , Kim MH , Hur S , Chung TM . WM–STGCN: a novel spatiotemporal modeling method for parkinsonian gait recognition. Sensors 2023;23(10):4980. 10.3390/s23104980 37430892 PMC10223022

[92] Zeng Q , Liu P , Yu N , Wu J , Huo W , Han J . Video‐based quantification of gait impairments in Parkinson's disease using skeleton‐Silhouette fusion convolution network. IEEE Trans Neural Syst Rehabil Eng 2023;31:2912–2922. 10.1109/TNSRE.2023.3291359 37418413

[93] Qin J , Liu Y , Cai M , Chen Y , Ji C , Wang Z . G2GNet: a model for video tremor diagnosis in Parkinson's disease. 2023 IEEE Smart World Congress (SWC) Piscataway, NJ: IEEE; 2023:1–6. 10.1109/SWC57546.2023.10449314.

[94] Liu P , Yu N , Yang Y , et al. Quantitative assessment of gait characteristics in patients with Parkinson's disease using 2D video. Parkinsonism Relat Disord 2022;101:49–56. 10.1016/j.parkreldis.2022.06.012 35793570

[95] Guo R , Li H , Zhang C , Qian X . A tree‐structure‐guided graph convolutional network with contrastive learning for the assessment of parkinsonian hand movements. Med Image Anal 2022;81:102560. 10.1016/j.media.2022.102560 35932545

[96] Morinan G , Peng Y , Rupprechter S , et al. Computer‐vision based method for quantifying rising from chair in Parkinson's disease patients. Intell‐Based Med 2022;6:100046. 10.1016/j.ibmed.2021.100046

[97] Monje MHG , Domínguez S , Vera‐Olmos J , et al. Remote evaluation of Parkinson's disease using a conventional webcam and artificial intelligence. Front Neurol 2021;12:742654. 10.3389/fneur.2021.742654 35002915 PMC8733479

[98] Shin JH , Yu R , Ong JN , et al. Quantitative gait analysis using a pose‐estimation algorithm with a single 2D‐video of Parkinson's disease patients. J Parkinsons Dis 2021;11(3):1271–1283. 10.3233/JPD-212544 33935106

[99] Park KW , Lee EJ , Lee JS , et al. Machine learning–based automatic rating for cardinal symptoms of Parkinson disease. Neurology 2021;96(13):e1761–e1769. 10.1212/WNL.0000000000011654 33568548

[100] Guo R , Sun J , Zhang C , Qian X . A contrastive graph convolutional network for toe‐tapping assessment in Parkinson's disease. IEEE Trans Circuits Syst Video Technol 2022;32(12):8864–8874. 10.1109/TCSVT.2022.3195854

[101] Guo R , Sun J , Zhang C , Qian X . A self‐supervised metric learning framework for the arising‐from‐chair assessment of parkinsonians with graph convolutional networks. IEEE Trans Circuits Syst Video Technol 2022;32(9):6461–6471. 10.1109/TCSVT.2022.3163959

[102] Hoang TH , Zallek C , Do MN . Smartphone‐based digitized neurological examination toolbox for multi‐test neurological abnormality detection and documentation. IEEE J Biomed Health Inform 2024;28(12):7457–7468. 10.1109/JBHI.2024.3439492 39186431

[103] Hsu YC , Su YH , Cheng BR , et al. Movement disorder evaluation of Parkinson's disease severity based on deep neural network models. IEEE Access 2024;12:143413–143433. 10.1109/ACCESS.2024.3436822

[104] Lai MH , Liu DG , Cheng CH , Lai CY . Quantitative assessment of gait in spinocerebellar ataxia using deep learning. 2024 IEEE 4th International Conference on Electronic Communications, Internet of Things and Big Data (ICEIB). Piscatawy, NJ: IEEE; 2024:40–43. 10.1109/ICEIB61477.2024.10602581.

[105] Yang Y , Liu P , Sun Y , Yu N , Wu J , Han J . A video‐based method to classify abnormal gait for remote screening of Parkinson's disease. 2021 40th Chinese Control Conference (CCC). Piscataway, NJ: IEEE; 2021:3357–3362. 10.23919/CCC52363.2021.9549992.

[106] Xie Z , Guo R , Zhang C , Qian X . A clinically guided graph convolutional network for assessment of parkinsonian pronation‐supination movements of hands. IEEE Trans Circuits Syst Video Technol 2024;34(5):3687–3699. 10.1109/TCSVT.2023.3318243

[107] Abe K , Tabei KI , Matsuura K , Kobayashi K , Ohkubo T . Relationship between the results of arm swing data from the OpenPose‐based gait analysis system and MDS‐UPDRS scores. IEEE Access 2022;10:118992–119000. 10.1109/ACCESS.2022.3220767

[108] Talitckii A , Kovalenko E , Shcherbak A , et al. Comparative study of wearable sensors, video, and handwriting to detect Parkinson's disease. IEEE Trans Instrum Meas 2022;71:1–10. 10.1109/TIM.2022.3176898

[109] Ajay J , Song C , Wang A , Langan J , Li Z , Xu W . A pervasive and sensor‐free deep learning system for parkinsonian gait analysis. 2018 IEEE EMBS International Conference on Biomedical & Health Informatics (BHI). Piscataway, NJ: IEEE; 2018:108–111. 10.1109/BHI.2018.8333381.

[110] Dentamaro V , Impedovo D , Pirlo G . Real‐time neurodegenerative disease video classification with severity prediction. In: Ricci E , Rota Bulò S , Snoek C , Lanz O , Messelodi S , Sebe N , eds. Image Analysis and Processing – ICIAP 2019. Cham, Switzerland: Springer International Publishing; 2019:618–628. 10.1007/978-3-030-30645-8_56.

[111] Nath T , Mathis A , Chen AC , Patel A , Bethge M , Mathis MW . Using DeepLabCut for 3D markerless pose estimation across species and behaviors. Nat Protoc 2019;14(7):2152–2176. 10.1038/s41596-019-0176-0 31227823

[112] Tajbakhsh N , Shin JY , Gurudu SR , et al. Convolutional neural networks for medical image analysis: full training or fine tuning? IEEE Trans Med Imaging 2016;35(5):1299–1312. 10.1109/TMI.2016.2535302 26978662

[113] Aravamuthan BR , Pearson TS , Ueda K , et al. Determinants of gait dystonia severity in cerebral palsy. Dev Med Child Neurol 2023;65(7):968–977. 10.1111/dmcn.15524 36701240 PMC10392706

[114] Nunes AS , Kozhemiako N , Stephen CD , Schmahmann JD , Khan S , Gupta AS . Automatic classification and severity estimation of ataxia from finger tapping videos. Front Neurol 2022;12:795258. 10.3389/fneur.2021.795258 35295715 PMC8919801

[115] Vignoud G , Desjardins C , Salardaine Q , et al. Video‐based automated assessment of movement parameters consistent with MDS‐UPDRS III in Parkinson's disease. J Parkinsons Dis 2022;12(7):2211–2222. 10.3233/JPD-223445 35964204 PMC9661322

[116] Williams S , Zhao Z , Hafeez A , et al. The discerning eye of computer vision: can it measure Parkinson's finger tap bradykinesia? J Neurol Sci 2020;416:117003. 10.1016/j.jns.2020.117003 32645513

[117] Vanmechelen I , Van Wonterghem E , Aerts JM , et al. Markerless motion analysis to assess reaching‐sideways in individuals with dyskinetic cerebral palsy: a validity study. J Biomech 2024;173:112233. 10.1016/j.jbiomech.2024.112233 39053292

[118] Bungay J , Emokpae O , Relton SD , et al. Contactless hand tremor amplitude measurement using smartphones: development and pilot evaluation. 2023 45th Annual International Conference of the IEEE Engineering in Medicine & Biology Society (EMBC). Piscataway, NJ: IEEE; 2023:1–4. 10.1109/EMBC40787.2023.10340420. 38083026

[119] Wang X , Garg S , Tran SN , Bai Q , Alty J . Hand tremor detection in videos with cluttered background using neural network based approaches. Health Inf Sci Syst 2021;9(1):30. 10.1007/s13755-021-00159-3 34276971 PMC8273850

[120] Peach R , Friedrich M , Fronemann L , et al. Head movement dynamics in dystonia: a multi‐centre retrospective study using visual perceptive deep learning. Npj Digit Med 2024;7(1):160. 10.1038/s41746-024-01140-6 38890413 PMC11189529

[121] Conelea C , Liang H , DuBois M , et al. Automated quantification of eye tics using computer vision and deep learning techniques. Mov Disord 2024;39(1):183–191. 10.1002/mds.29593 38146055 PMC10895867

[122] Brügge NS , Sallandt GM , Schappert R , et al. Automated motor tic detection: a machine learning approach. Mov Disord 2023;38(7):1327–1335. 10.1002/mds.29439 37166278

[123] Zhao Y , Liu Y , Li J , et al. Global joint information extraction convolution neural network for Parkinson's disease diagnosis. Expert Syst Appl 2024;243:122837. 10.1016/j.eswa.2023.122837

[124] Li Z , Lu K , Cai M , Liu X , Wang Y , Yang J . An automatic evaluation method for Parkinson's dyskinesia using finger tapping video for small samples. J Med Biol Eng 2022;42(3):351–363. 10.1007/s40846-022-00701-y

[125] Oku T , Furuya S , Lee A , Altenmüller E . Video‐based diagnosis support system for pianists with Musician's dystonia. Front Neurol 2024;15:1409962. 10.3389/fneur.2024.1409962 39015318 PMC11250081

[126] Guarín DL , Wong JK , McFarland NR , Ramirez‐Zamora A . Characterizing disease progression in Parkinson's disease from videos of the finger tapping test. IEEE Trans Neural Syst Rehabil Eng Publ IEEE Eng Med Biol Soc 2024;32:2293–2301. 10.1109/TNSRE.2024.3416446 PMC11260436 38905096

[127] Verdonck T , Baesens B , Óskarsdóttir M , vanden Broucke S . Special issue on feature engineering editorial. Mach Learn 2024;113(7):3917–3928. 10.1007/s10994-021-06042-2

[128] Dzieżyc M , Gjoreski M , Kazienko P , Saganowski S , Gams M . Can we ditch feature engineering? End‐to‐end deep learning for affect recognition from physiological sensor data. Sensors 2020;20(22):6535. 10.3390/s20226535 33207564 PMC7697590

[129] Ronneberger O , Fischer P , Brox T . U‐Net: Convolutional Networks for Biomedical Image Segmentation; arXiv.org. Published online May 2015. Accessed September 27, 2024. https://arxiv.org/abs/1505.04597v1.

[130] Yang J , Williams S , Hogg DC , Alty JE , Relton SD . Deep learning of Parkinson's movement from video, without human‐defined measures. J Neurol Sci 2024;463:123089. 10.1016/j.jns.2024.123089 38991323

[131] Archila J , Manzanera A , Martínez F . A multimodal Parkinson quantification by fusing eye and gait motion patterns, using covariance descriptors, from non‐invasive computer vision. Comput Methods Programs Biomed 2022;215:106607. 10.1016/j.cmpb.2021.106607 34998167

[132] Liang A . Assessing gait dysfunction severity in Parkinson's disease using 2‐stream spatial‐temporal neural network. J Biomed Inform 2024;157:104679. 10.1016/j.jbi.2024.104679 38925280

[133] Silvis‐Cividjian N . Therac‐25 accidents: we keep on learning from them. Computertomographie 2024;57(12):69–78. 10.1109/MC.2024.3450197

[134] Zhang H , Ho ESL , Zhang FX , Del Din S , Shum HPH . Pose‐based tremor type and level analysis for Parkinson's disease from video. Int J Comput Assist Radiol Surg 2024;19(5):831–840. 10.1007/s11548-023-03052-4 38238490 PMC11098891

[135] Ali MR , Sen T , Li Q , et al. Analyzing head pose in remotely collected videos of people with Parkinson's disease. ACM Trans Comput Healthc 2021;2(4):30:1–30:13. 10.1145/3459669

[136] Paulus T , Schappert R , Bluschke A , et al. Questioning the definition of Tourette syndrome—evidence from machine learning. Brain Commun 2021;3(4):fcab282. 10.1093/braincomms/fcab282 34993475 PMC8728701

[137] Simonyan K , Vedaldi A , Zisserman A . Deep inside convolutional networks: Visualising image classification models and saliency maps; Published online April 2014. 10.48550/arXiv.1312.6034.

[138] Lundberg SM , Lee SI . A unified approach to interpreting model predictions. Advances in Neural Information Processing Systems. Vol. 30. New York: Curran Associates, Inc.; 2017 Accessed October 16, 2024. https://papers.nips.cc/paper_files/paper/2017/hash/8a20a8621978632d76c43dfd28b67767-Abstract.html.

[139] Ma C , Guo L , Pan L , et al. Tremor detection transformer: an automatic symptom assessment framework based on refined whole‐body pose estimation. Eng Appl Artif Intel 2023;125:106645. 10.1016/j.engappai.2023.106645

[140] Rahman W , Hasan M , Islam MS , et al. Auto‐gait: automatic ataxia risk assessment with computer vision from gait task videos. Proc ACM Interact Mob Wearable Ubiquitous Technol 2023;7(1):26:1–26:19. 10.1145/3580845 39558922 PMC11571898

[141] Yang N , Liu DF , Liu T , et al. Automatic detection pipeline for accessing the motor severity of Parkinson's disease in finger tapping and postural stability. IEEE Access 2022;10:66961–66973. 10.1109/ACCESS.2022.3183232

[142] Ferri C , Hernández‐Orallo J , Modroiu R . An experimental comparison of performance measures for classification. Pattern Recognit Lett 2009;30(1):27–38. 10.1016/j.patrec.2008.08.010

[143] Maier‐Hein L , Reinke A , Godau P , et al. Metrics reloaded: recommendations for image analysis validation. Nat Methods 2024;21(2):195–212. 10.1038/s41592-023-02151-z 38347141 PMC11182665

[144] Pintea SL , Zheng J , Li X , et al. Hand‐tremor frequency estimation in videos. In: Leal‐Taixé L , Roth S , eds. Computer Vision – ECCV 2018 Workshops. Cham, Switzerland: Springer International Publishing; 2019:213–228. 10.1007/978-3-030-11024-6_14.

[145] Zhao Y , Zhao L , Zhou X , et al. Distilling vision‐language models on millions of videos; Published online April 15, 2024 10.48550/arXiv.2401.06129.

[146] Zhang Z , Meng W , Song M , et al. Application of multi‐angle millimeter‐wave radar detection in human motion behavior and micro‐action recognition. Meas Sci Technol 2022;33(10):105107. 10.1088/1361-6501/ac7779

[147] Sun Z , Ke Q , Rahmani H , Bennamoun M , Wang G , Liu J . Human action recognition from various data modalities: a review. IEEE Trans Pattern Anal Mach Intell 2023;45(3):3200–3225. 10.1109/TPAMI.2022.3183112 35700242

[148] Hao Z , Niu J , Dang X , Qiao Z . WiPg: contactless action recognition using ambient Wi‐fi signals. Sensors. 2022;22(1):402. 10.3390/s22010402 35009943 PMC8749714

[149] Dong Y , Li Y , Zhao D , Shen G , Zeng Y . Bullying10K: a large‐scale neuromorphic dataset towards privacy‐preserving bullying recognition. Adv Neural Inf Process Syst 2023;36:1923–1937.

[150] Spann J , Chen SA , Ashizawa T , Hoque E . Getting on the right foot: using observational and quantitative methods to evaluate movement disorders. Proceedings of the 29th International Conference on Intelligent User Interfaces. IUI ‘24. New York: Association for Computing Machinery; 2024:742–749. 10.1145/3640543.3645160.

[151] Tang W , van Ooijen PMA , Sival DA , Maurits NM . Classification of movement disorders using video recordings of gait with attention‐based graph convolutional networks. 2023 IEEE EMBS International Conference on Biomedical and Health Informatics (BHI). Piscataway, NJ: IEEE; 2023:1–4. 10.1109/BHI58575.2023.10313397.

[152] Zhou Y , Xiao L , Su X , Xu X . Assessment of Parkinson's motor severity by multi‐feature and multi‐scale motion convolutional neural network with positional encoding. 2023 4th International Seminar on Artificial Intelligence, Networking and Information Technology (AINIT). Piscataway, NJ: IEEE; 2023:179–183. 10.1109/AINIT59027.2023.10212599.

[153] Sabo A , Iaboni A , Taati B , Fasano A , Gorodetsky C . Evaluating the ability of a predictive vision‐based machine learning model to measure changes in gait in response to medication and DBS within individuals with Parkinson's disease. Biomed Eng Online 2023;22:120. 10.1186/s12938-023-01175-y 38082277 PMC10714555

[154] Yu T , Park KW , McKeown MJ , Wang ZJ . Clinically informed automated assessment of finger tapping videos in Parkinson's disease. Sensors. 2023;23(22):9149. 10.3390/s23229149 38005535 PMC10674854

[155] Morgan C , Masullo A , Mirmehdi M , et al. Automated real‐world video analysis of sit‐to‐stand transitions predicts Parkinson's disease severity. Digit Biomark 2023;7(1):92–103. 10.1159/000530953 37588481 PMC10425718

[156] Li G , Pun CM , Li H , Xiong J , Xu F , Gao H . An Optimized‐Skeleton‐Based Parkinsonian Gait Auxiliary Diagnosis Method with Both Monitoring Indicators and Assisted Ratings. 2023 IEEE International Conference on Bioinformatics and Biomedicine (BIBM). Piscataway, NJ: IEEE; 2023:2011–2016. 10.1109/BIBM58861.2023.10385700.

[157] Wu J , Zhou T , Guo Y , et al. Video‐based evaluation system for tic action in Tourette syndrome: modeling, detection, and evaluation. Health Inf Sci Syst 2023;11(1):39. 10.1007/s13755-023-00240-z 37649855 PMC10462598

[158] Azami H , Chang Z , Arnold SE , Sapiro G , Gupta AS . Detection of oculomotor dysmetria from Mobile phone video of the horizontal saccades task using signal processing and machine learning approaches. IEEE Access Pract Innov Open Solut 2022;10:34022. 10.1109/access.2022.3156964 PMC9632643 36339795

[159] Tang W , van Ooijen PMA , Sival DA , Maurits NM . 2D gait skeleton data normalization for quantitative assessment of movement disorders from freehand single camera video recordings. Sensors 2022;22(11):4245. 10.3390/s22114245 35684866 PMC9185346

[160] Vu JP , Cisneros E , Lee HY , et al. Head tremor in cervical dystonia: quantifying severity with computer vision. J Neurol Sci 2022;434:120154. 10.1016/j.jns.2022.120154 35101766 PMC9059761

[161] Zhang Z , Cisneros E , Lee HY , et al. Hold that pose: capturing cervical dystonia's head deviation severity from video. Ann Clin Transl Neurol 2022;9(5):684–694. 10.1002/acn3.51549 35333449 PMC9082391

[162] Novotny M , Tykalova T , Ruzickova H , Ruzicka E , Dusek P , Rusz J . Automated video‐based assessment of facial bradykinesia in de‐novo Parkinson's disease. NPJ Digit Med 2022;5:98. 10.1038/s41746-022-00642-5 35851859 PMC9293947

[163] Zhao A , Li J . Two‐channel lstm for severity rating of parkinson's disease using 3d trajectory of hand motion. Multimed Tools Appl 2022;81(23):33851–33866. 10.1007/s11042-022-12659-9

[164] Geng F , Ding Q , Wu W , et al. Light‐efficient channel attention in convolutional neural networks for tic recognition in the children with tic disorders. Front Comput Neurosci 2022;16:1047954. 10.3389/fncom.2022.1047954 36405786 PMC9673168

[165] Nachum R , Jackson K , Duric Z , Gerber L . A Novel Computer Vision Approach to Kinematic Analysis of Handwriting with Implications for Assessing Neurodegenerative Diseases. 2021 43rd Annual International Conference of the IEEE Engineering in Medicine & Biology Society (EMBC). Piscataway, NJ: IEEE; 2021:1309–1313. 10.1109/EMBC46164.2021.9630492. 34891526

[166] Lu M , Zhao Q , Poston KL , et al. Quantifying Parkinson's disease motor severity under uncertainty using MDS‐UPDRS videos. Med Image Anal 2021;73:102179. 10.1016/j.media.2021.102179 34340101 PMC8453121

[167] Guo R , Shao X , Zhang C , Qian X . Multi‐scale sparse graph convolutional network for the assessment of parkinsonian gait. IEEE Trans Multimed 2022;24:1583–1594. 10.1109/TMM.2021.3068609

[168] Mehta D , Asif U , Hao T , et al. Towards automated and marker‐less Parkinson disease assessment: predicting UPDRS scores using sit‐stand videos. 2021 IEEE/CVF Conference on Computer Vision and Pattern Recognition Workshops (CVPRW). Piscataway, NJ: IEEE; 2021:3836–3844. 10.1109/CVPRW53098.2021.00425.

[169] Yin Z , Geraedts VJ , Wang Z , Contarino MF , Dibeklioglu H , van Gemert J . Assessment of Parkinson's disease severity from videos using deep architectures. IEEE J Biomed Health Inform 2022;26(3):1164–1176. 10.1109/JBHI.2021.3099816 34310333

[170] Khan T , Zeeshan A , Dougherty M . A novel method for automatic classification of Parkinson gait severity using front‐view video analysis. Technol Health Care 2021;29(4):643–653. 10.3233/THC-191960 33427697 PMC9789477

[171] Williams S , Relton SD , Fang H , et al. Supervised classification of bradykinesia in Parkinson's disease from smartphone videos. Artif Intell Med 2020;110:101966. 10.1016/j.artmed.2020.101966 33250146

[172] Ali MR , Hernandez J , Dorsey ER , Hoque E , McDuff D . Spatio‐temporal attention and magnification for classification of Parkinson's disease from videos collected via the internet. 2020 15th IEEE International Conference on Automatic Face and Gesture Recognition (FG 2020). Piscataway, NJ: IEEE; 2020:207–214. 10.1109/FG47880.2020.00008.

[173] Hu K , Wang Z , Mei S , et al. Vision‐based freezing of gait detection with anatomic directed graph representation. IEEE J Biomed Health Inform 2020;24(4):1215–1225. 10.1109/JBHI.2019.2923209 31217134

[174] Lu M , Poston K , Pfefferbaum A , et al. Vision‐based estimation of MDS‐UPDRS gait scores for assessing Parkinson's disease motor severity. In: Martel AL , Abolmaesumi P , Stoyanov D , et al., eds. Medical Image Computing and Computer Assisted Intervention – MICCAI 2020. Cham, Switzerland: Springer International Publishing; 2020:637–647. 10.1007/978-3-030-59716-0_61. PMC7585545 33103164

[175] Guo R , Shao X , Zhang C , Qian X . Sparse adaptive graph convolutional network for leg agility assessment in Parkinson's disease. IEEE Trans Neural Syst Rehabil Eng Publ IEEE Eng Med Biol Soc 2020;28(12):2837–2848. 10.1109/TNSRE.2020.3039297 33211661

[176] Chang Z , Chen Z , Stephen CD , et al. Accurate detection of cerebellar smooth pursuit eye movement abnormalities via mobile phone video and machine learning. Sci Rep 2020;10:18641. 10.1038/s41598-020-75661-x 33122811 PMC7596555

[177] Langevin R , Ali MR , Sen T , et al. The PARK framework for automated analysis of Parkinson's disease characteristics. Proc ACM Interact Mob Wearable Ubiquitous Technol 2019;3(2):54:1–54:22. 10.1145/3328925

[178] Wong DC , Relton SD , Fang H , et al. Supervised classification of bradykinesia for Parkinson's disease diagnosis from smartphone videos. 2019 IEEE 32nd International Symposium on Computer‐Based Medical Systems (CBMS). Piscataway, NJ: IEEE; 2019:32–37. 10.1109/CBMS.2019.00017.

[179] Liu Y , Chen J , Hu C , et al. Vision‐based method for automatic quantification of parkinsonian bradykinesia. IEEE Trans Neural Syst Rehabil Eng 2019;27(10):1952–1961. 10.1109/TNSRE.2019.2939596 31502982

[180] Cuppens K , Chen CW , Wong KBY , et al. Using spatio‐temporal interest points (STIP) for myoclonic jerk detection in nocturnal video. 2012 Annual International Conference of the IEEE Engineering in Medicine and Biology Society. Piscataway, NJ: IEEE; 2012:4454–4457. 10.1109/EMBC.2012.6346955. 23366916

[181] Chen YY , Cho CW , Lin SH , et al. A vision‐based regression model to evaluate parkinsonian gait from monocular image sequences. Expert Syst Appl 2012;39(1):520–526. 10.1016/j.eswa.2011.07.042

[182] Chen SW , Lin SH , Liao LD , et al. Quantification and recognition of parkinsonian gait from monocular video imaging using kernel‐based principal component analysis. Biomed Eng Online 2011;10:99. 10.1186/1475-925X-10-99 22074315 PMC3354347

[183] Ho MY , Kuo MC , Chen CS , et al. Pathological gait analysis with an open‐source cloud‐enabled platform empowered by semi‐supervised learning‐PathoOpenGait. IEEE J Biomed Health Inform 2024;28(2):1066–1077. 10.1109/JBHI.2023.3340716 38064333

[184] Shin J , Matsumoto M , Maniruzzaman M , et al. Classification of hand‐movement disabilities in Parkinson's disease using a motion‐capture device and machine learning. IEEE Access 2024;12:52466–52479. 10.1109/ACCESS.2024.3386367

[185] Amprimo G , Rechichi I , Ferraris C , Olmo G . Objective assessment of the finger tapping task in Parkinson's disease and control subjects using azure Kinect and machine learning. 2023 IEEE 36th International Symposium on Computer‐Based Medical Systems (CBMS). Piscataway, NJ: IEEE; 2023:640–645. 10.1109/CBMS58004.2023.00293.

[186] Bama S , Jinila B . Vision‐based gait analysis for real‐time Parkinson disease identification and diagnosis system. Health Syst 2022;13(1):62–72. 10.1080/20476965.2022.2125838 PMC11687389 39741664

[187] Sabo A , Mehdizadeh S , Iaboni A , Taati B . Estimating parkinsonism severity in natural gait videos of older adults with dementia. IEEE J Biomed Health Inform 2022;26(5):2288–2298. 10.1109/JBHI.2022.3144917 35077373

[188] Summa S , Tartarisco G , Favetta M , et al. Validation of low‐cost system for gait assessment in children with ataxia. Comput Methods Programs Biomed 2020;196:105705. 10.1016/j.cmpb.2020.105705 32846316

[189] Moshkova A , Samorodov A , Voinova N , Volkov A , Ivanova E , Fedotova E . Parkinson's Disease Detection by Using Machine Learning Algorithms and Hand Movement Signal from LeapMotion Sensor. 2020 26th Conference of Open Innovations Association (FRUCT). Piscataway, NJ: IEEE; 2020:321–327. 10.23919/FRUCT48808.2020.9087433.

[190] Reyes JF , Steven Montealegre J , Castano YJ , Urcuqui C , Navarro A . LSTM and Convolution Networks exploration for Parkinson's Diagnosis. 2019 IEEE Colombian Conference on Communications and Computing (COLCOM). Piscataway, NJ: IEEE; 2019:1–4. 10.1109/ColComCon.2019.8809160.

[191] Vivar G , Almanza‐Ojeda DL , Cheng I , Gomez JC , Andrade‐Lucio JA , Ibarra‐Manzano MA . Contrast and homogeneity feature analysis for classifying tremor levels in Parkinson's disease patients. Sensors 2019;19(9):2072. 10.3390/s19092072 31060214 PMC6539600

[192] Butt AH , Rovini E , Dolciotti C , et al. Objective and automatic classification of Parkinson disease with leap motion controller. Biomed Eng Online 2018;17(1):168. 10.1186/s12938-018-0600-7 30419916 PMC6233603

[193] Urcuqui C , Castaño Y , Delgado J , et al. Exploring machine learning to analyze Parkinson's disease patients. 2018 14th International Conference on Semantics, Knowledge and Grids (SKG). Piscataway, NJ: IEEE; 2018:160–166. 10.1109/SKG.2018.00029.

[194] Soltaninejad S , Rosales‐Castellanos A , Ba F , Ibarra‐Manzano MA , Cheng I . Body movement monitoring for Parkinson's disease patients using a smart sensor based non‐invasive technique. 2018 IEEE 20th International Conference on E‐Health Networking, Applications and Services (Healthcom). Piscataway, NJ: IEEE; 2018:1–6. 10.1109/HealthCom.2018.8531197.

[195] Li Q , Wang Y , Sharf A , et al. Classification of gait anomalies from kinect. Vis Comput 2018;34(2):229–241. 10.1007/s00371-016-1330-0

[196] Dror B , Yanai E , Frid A , et al. Automatic assessment of Parkinson's disease from natural hands movements using 3D depth sensor. 2014 IEEE 28th Convention of Electrical & Electronics Engineers in Israel (IEEEI). Piscataway, NJ: IEEE; 2014:1–5. 10.1109/EEEI.2014.7005763.

[197] Dyshel M , Arkadir D , Bergman H , Weinshall D . Quantifying Levodopa‐Induced Dyskinesia Using Depth Camera. 2015 IEEE International Conference on Computer Vision Workshop (ICCVW). Piscataway, NJ: IEEE; 2015:511–518. 10.1109/ICCVW.2015.73.

[198] Ye C , Xiao Y , Li R , et al. Pilot feasibility study of a multi‐view vision based scoring method for cervical dystonia. Sensors 2022;22(12):4642. 10.3390/s22124642 35746424 PMC9230118

[199] Buongiorno D , Bortone I , Cascarano GD , Trotta GF , Brunetti A , Bevilacqua V . A low‐cost vision system based on the analysis of motor features for recognition and severity rating of Parkinson's disease. BMC Med Inform Decis Mak 2019;19(9):243. 10.1186/s12911-019-0987-5 31830986 PMC6907109

[200] Ferraris C , Nerino R , Chimienti A , et al. A self‐managed system for automated assessment of UPDRS upper limb tasks in Parkinson's disease. Sensors 2018;18(10):3523. 10.3390/s18103523 30340420 PMC6210162

[201] Lugaresi, C , Tang, J , Nash, H et al MediaPipe: a framework for building perception pipelines. Published online June 2019. 10.48550/arXiv.1906.08172

[202] Mathis A , Mamidanna P , Cury KM , et al. DeepLabCut: markerless pose estimation of user‐defined body parts with deep learning. Nat Neurosci 2018;21(9):1281–1289. 10.1038/s41593-018-0209-y 30127430

